# Optimizing the Readout of Lanthanide-DOTA Complexes for the Detection of Ligand-Bound Copper(I)

**DOI:** 10.3390/molecules22050802

**Published:** 2017-05-14

**Authors:** Jill R. Hanna, Christopher Allan, Charlotte Lawrence, Odile Meyer, Neil D. Wilson, Alison N. Hulme

**Affiliations:** EaStCHEM School of Chemistry, The University of Edinburgh, Joseph Black Building, David Brewster Road, Edinburgh EH9 3FJ, UK; s0789206@ed-alumni.net (J.R.H.); C.Allan-3@sms.ed.ac.uk (C.A.); s0569419@ed-alumni.net (C.L.); odile.meyer1@ac-strasbourg.fr (O.M.); s0094548@ed-alumni.net (N.D.W.)

**Keywords:** CuAAC click reaction, DOTA complexes, luminescence, Cu(I) sensor, picolinyl azides, coumarin azides

## Abstract

The CuAAC ‘click’ reaction was used to couple alkyne-functionalized lanthanide-DOTA complexes to a range of fluorescent antennae. Screening of the antenna components was aided by comparison of the luminescent output of the resultant sensors using data normalized to account for reaction conversion as assessed by IR. A maximum 82-fold enhanced signal:background luminescence output was achieved using a Eu(III)-DOTA complex coupled to a coumarin-azide, in a reaction which is specific to the presence of copper(I). This optimized complex provides a new lead design for lanthanide-DOTA complexes which can act as irreversible ‘turn-on’ catalytic sensors for the detection of ligand-bound copper(I).

## 1. Introduction

The misregulation of copper in humans is linked with serious neurodegenerative disorders such as Menkes and Wilson’s diseases [1,2,3], amyotrophic lateral sclerosis [4,5], and Alzheimer’s disease [6,7]. To avoid the accumulation of excess copper, cells control copper pools rigorously through a range of uptake, storage, and trafficking mechanisms; this maintains a low concentration of labile copper ions, while simultaneously preserving sufficient cellular copper stores for function. It is thought that there are essentially no ‘free’ copper ions within the cell [8]. Although, there has been considerable progress in the development of fluorescent sensors for both copper(I) and copper(II) in recent years [9,10], most of these sensors rely on the detection of copper(I) through complexation of the ‘free’ metal ion giving rise to a concomitant change in spectroscopic properties of the sensor molecule in either a turn-on or turn-off sense. In an alternative strategy, we [11] and others [12] have reported reaction-based probes [13], which rely upon the irreversible formation or breaking of covalent bonds rather than the formation of supramolecular complexes. These ‘reactive’ probes can be used to detect the presence of ligand-bound copper(I); however, to date these sensors have been limited by either a modest signal output (10-fold increase in signal over background) [11], or the limit of detection (20 μM) [12]. Clearly, the signal output which a turn-on catalytic sensor must generate in order to be ‘useful’ depends upon the specific application, but in the current study we set a goal of attaining a signal enhancement of 10^2^–10^3^ fold.

**Figure 1:**
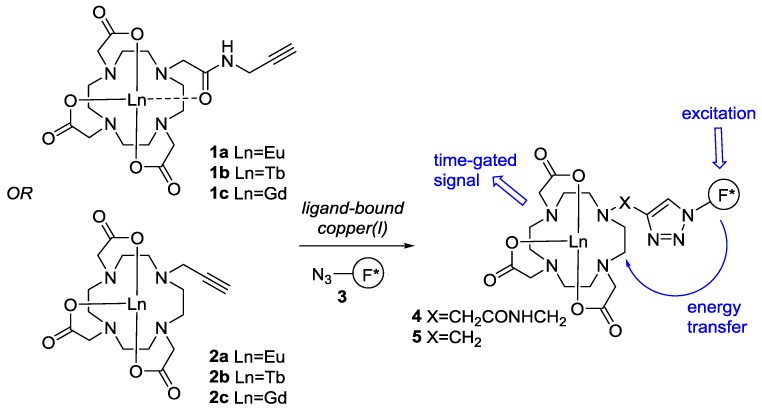
Sensor design for the detection of ligand-bound copper(I).

Lanthanide chelates offer considerable advantages over the use of standard fluorescent dyes for detection in vivo, especially when there is significant autofluorescence [14]. Our sensor design (Figure 1) relies upon the copper(I)-catalyzed azide alkyne cycloaddition (CuAAC) coupling of two components—a lanthanide-DOTA complex **1** or **2**, and a fluorescent azide antenna **3**—to give a luminescent complex **4** or **5**. Since we [11,15] and others [16,17,18,19] first introduced the use of bioorthogonal DOTA-alkyne complexed lanthanides such as **1** and **2** for ‘click’ chemistry they have found a range of applications; from metal ion reporting [11,20], to MR contrast agents [16,17,21,22,23]. It was envisaged that a dramatic improvement in their application to copper(I) sensing might be achieved through optimization of the energy transfer process. This might be achieved through: (i) screening of the fluorescent azides **3** used as the antenna; (ii) an investigation of the lanthanides at the core of the sensor itself (**1a** Ln = Eu, **1b** Ln = Tb etc.); (iii) alteration of the linker length between the DOTA core and the fluorescent antenna (e.g., using DOTA-alkyne complexes **1a**, **2a**, etc.); and (iv) reversal of the component reactivity, i.e., DOTA-azide complexes reacting with fluorescent alkynes (not shown). Because the CuAAC reaction of DOTA complexes such as **2a** and **2b** has been reported to be sluggish [18,19,24], rendering them unsuitable for use as sensors, we chose to focus this study on approaches (i) and (ii).

## 2. Results and Discussion

### 2.1. Synthesis of Sensor Components

**Figure 2:**
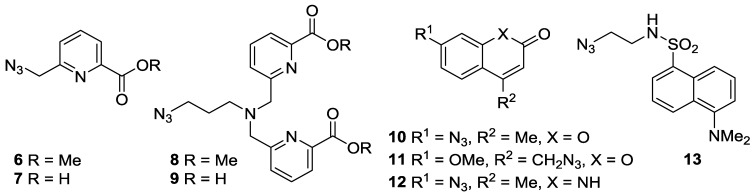
Fluorescent azides screened in this study.

Lanthanide DOTA complexes **1a** and **1b** were prepared by standard methods through functionalization of 1,4,7,10-tetraazacyclododecane-4,7,10-triacetic acid tri-*tert*-butyl ester (DO3*^t^*Bu) [25] with propargyl chloroacetamide [26], followed by deprotection of the acid functionalities and complexation with either Eu(OTf)_3_ or Tb(OTf)_3_ (Supplementary Material (SM) Scheme S1) [11]. Although lanthanides have previously been shown to be sensitised by a variety of fluorophores [27,28,29,30,31], with a view to potential future biological applications of our sensor, we restricted ourselves to four classes of fluorophore azide (**3**) which have been shown to be cell-permeable and to have potential in two-photon excitation studies (Figure 2).

1. Picolinate-derivatized ligands have previously been shown to act as sensitizers for europium and terbium ions [32,33,34,35], to undergo cellular entry via diffusion, and to be suitable for two photon excitation studies. Thus picolinate azides **6**–**9** were prepared from pyridine-2,6-dicarboxylic acid dimethyl ester through ready adaptation of the synthetic route to the 10-coordinate *N*,*N*,*N*′,*N*′-tetrakis[(6-carboxypyridin-2-yl)methyl]ethylenediamine (tpaen) ligand reported by Mazzanti et al. [33,36,37] (SM Scheme S2; three steps (28% overall), four steps (15% overall), three steps (27% overall), and four steps (22% overall) respectively).
2. Lanthanide complexes based on coumarin derivatives were pursued due to the known membrane permeability of coumarin azides [38], and previous reports of strong fluorescence activation of lanthanides by coumarin [39,40,41,42]. Coumarin azides **10** [43] and **11** [44] were both readily prepared in one step from commercially available starting materials (in 72% and 82% yields, respectively).
3. A derivative of carbostyril 124, azide **12**, was chosen because lanthanides complexed with ligands functionalised with carbostyril 124 have been shown to have long-lifetimes, good water solubility, and measurable brightness [45,46,47]. Diazotization of carbostyril 124, followed by addition of sodium azide, yielded **12** (65%) [48].
4. Finally, for comparison with our previously reported CuAAC reaction [11], dansyl azide **13** was prepared in two steps from dansyl chloride (70% overall) [49].

### 2.2. Component Coupling by the CuAAC Reaction

In our original work we made use of glutathione ligands for the CuAAC reaction, as complexation of Cu(I) to the carboxylate anion of glutathione (GS^−^), to give a GS^−^-Cu(I) complex, is thought to provide a pooling mechanism for Cu(I) in living cells [11,12]. However, this complex was found to be quite sensitive to environmental conditions and, in order to conduct a component screen, alternative catalyst systems were sought. The CuAAC reaction may be catalyzed by a range of ligand-bound copper(I) sources [50,51,52]. Notably, the readily accessible, stable ligand *tris*-(benzyltriazolemethyl)amine (TBTA) has been shown to bind strongly to the copper(I) ion [53], making it a good model for ligand-bound copper(I). Thus, to screen for alkyne/azide pairings with the most intense luminescent read-out, lanthanide-alkyne complex **1a** or **1b** was premixed with TBTA in *^t^*BuOH:H_2_O (2:1), sodium ascorbate was added followed by copper(II) sulfate, finally the fluorescent azide (1 equivalent) was added and the mixture stirred at room temperature for 16 h. To quench the reaction, the solution was gently shaken with a metal scavenger resin to remove the copper. Removal of both the scavenger resin and solvent gave crude products in which the formation of the luminescent DOTA complex could be confirmed by ESI-MS.

The optimum wavelength for excitation of each sensor complex was determined from the excitation spectrum of the fluorophore component (Table 1). Dilution experiments (performed on **19-Eu**) indicated that a standard concentration of 100 μM in water would give the sharpest luminescence emission spectra. The europium luminescence arising from ^5^D_o_ to ^7^F_J_ transitions was evaluated at 593 or 615 nm, depending on which offered the greatest increase in signal; whilst terbium luminescence was measured at 545 nm as this provides the most intense terbium transition.

### 2.3. Initial Analysis of the Sensor Design by Component

**Figure 3:**
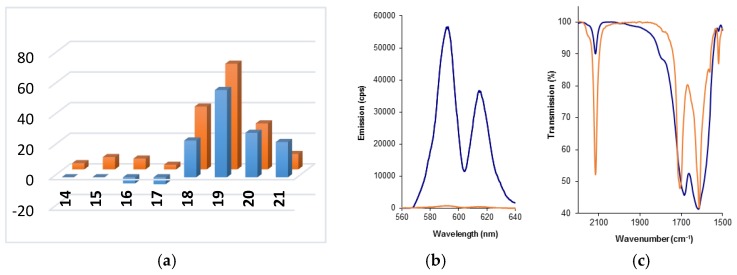
Change in Signal Output after CuAAC Reaction: (**a**) Sensor-Ln complex (Front (blue): Tb; Back (orange): Eu) vs. Signal increase over background (fold); (**b**) Luminescence of crude sensor **19-Eu** (blue) and background **1a**:**11** (1:1) (orange), showing a 69-fold increase in emission (measured at 100 μM in H_2_O, time delay = 0.076 ms, slits = 10 nm, sample window = 5 ms, number of flashes = 20); (**c**) FT-IR analysis of azide peak at 2100 cm^−1^ for crude sensor **19-Eu** (blue) with normalization of absorbance to carbonyl band at 1612 cm^−1^ in a 1:1 mixture of **1a**:**11** (orange).

To facilitate comparison of the different sensors, the increase in lanthanide luminescence intensity between a background spectrum (1:1 mix of alkyne and azide components, 100 μM in water) and the crude lanthanide sensor was expressed as a “fold increase” (Figure 3a,b, SM Tables S1–S4). These data indicate that—in terms of both the absolute signal brightness, and fold-increase over background—sensor **19-Eu**, arising from the CuAAC coupling of DOTA-alkyne complex **1a** with coumarin azide **11**, offers the optimum readout. These experiments also highlighted that CuAAC coupling of picolinate-derived ligands to DOTA-alkyne complex **1a** gave complexes (**14**-**17**)-**Eu** in which the europium was not efficiently sensitised. This is perhaps not surprising, because it has already been shown that, in general, sensitization of terbium by picolinate-derived ligands is more efficient [33]. However in the case of complexes **16-Tb** and **17-Tb** (which combine terbium alkyne complex **1b** with the di-picolinate ligands **8** and **9**), surprisingly high background signals were observed for the 1:1 mix of the azide and alkyne components, accompanied by a significant decrease in signal (4–4.5-fold) on CuAAC coupling of the components. This suggests that the component azides **8** and **9** actually bind to, and sensitise, the terbium metal rather well; but that when they are separated from the terbium ion through CuAAC coupling to the DOTA core, this sensitization is significantly reduced.

### 2.4. Normalization of Luminescence Output Data by IR

In comparing the output data, it became clear that not all of the reactions had reached completion within the standard 16 h timeframe used for the CuAAC coupling reaction. This was evidenced by MS data which indicated the presence of both starting material(s) and coupled product (e.g., SM Figure S1 for **20-Tb**), and is not surprising given the use of TBTA which is known to have relatively poor acceleration characteristics in the CuAAC reaction [50,51,52]. In seeking a rapid analysis method to determine the absolute increase in signal for each coupled product over the background signal, we were attracted to the use of vibrational spectroscopies such as IR to determine the extent to which the reaction had proceeded in the crude reaction mixture. In situ IR monitoring, using the azide absorption at ~2100 cm^−1^ for quantification, has previously been used to reveal mechanistic insights into the CuAAC reaction [54]. The utility of this technique to determine click reaction conversions, based on IR absorption levels corresponding to residual azide, has also been reported in a variety of contexts [55,56,57,58,59,60]. IR monitoring is particularly appropriate in this instance, as both the sensor complex and the lanthanide-DOTA core are paramagnetic; rendering reaction monitoring and assessment of product purity by NMR challenging.

**Figure 4:**
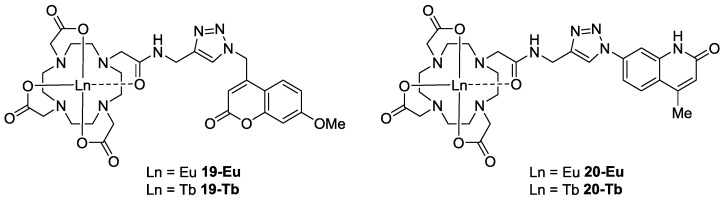
Structures of lead sensors **19-Eu**/**Tb** and **20-Eu**/**Tb**.

The presence of carbonyl bands in both the initial complex (**1a** or **1b**) and the CuAAC coupled product (**4**, Figure 1), which were not expected to shift in either position or intensity following the CuAAC reaction, led to an expectation that normalization and hence quantification of the conversion data could be achieved [56,58]. In order to demonstrate that there were no unexpected absorption peaks in the azide region of the coupled product, a sample of one of the complexes was purified by HPLC (SM Figure S2 for **19-Tb**); the IR spectrum of the purified material showed the complete absence of signal at 2115 cm^−1^ which had previously been ascribed to the presence of unreacted azide in the crude material. In addition, experiments with the comparatively unreactive Eu-DOTA complex **2a** showed that, under identical CuAAC conditions when there is no coupling reaction, the fluorophore azide absorptions remain at their original intensities. On this basis, IR spectra for the crude product mixtures from four of the most promising reactions were normalized (using the carbonyl bands indicated in Table 2) against composite spectra generated for the appropriate 1:1 mixtures of initial azide and alkyne components (Figure 3c, SM Tables S5 and S6). The percentage of unreacted azide remaining in the crude product mixtures was determined and was used to estimate the percentage conversion of each of these reactions. These conversion values were then used to adjust the maximal emission output of the lead sensors (Figure 4) to allow rapid determination of the ‘optimum’ sensor design; the maximal output for sensor **19-Eu** was determined to be an 82-fold increase over background using this normalization process. This lead complex was purified by HPLC (SM Figure S3) and its UV-vis (SM Figure S4), MS (SM Figure S5) and ^1^H-NMR (SM) spectra were acquired.

### 2.5. Metal Ion Specificity for Formation of the Optimum Complex ***19-Eu***

Metal ion specificity for the optimum sensor was confirmed through coupling of DOTA alkyne **1a** and coumarin azide **11** to give **19-Eu** in three solutions, one containing Na(I), K(I), Fe(II), Ni(II), Zn(II), and Cu(II) (10 mol% each); a second containing CuSO_4_ (10 mol%), sodium ascorbate (20 mol%) and TBTA (10 mol%); and a third in which there were no metal ions present. Each solution was stirred for 16 h at room temperature and, following metal ion extraction as described in Section 2.2, was excited at 325 nm. The lanthanide luminescence was measured at 593 nm; the mixed ion solution (without Cu(I)) showed no increase in signal over background (at ~800 cps), whilst the reaction conducted in the presence of Cu(I) showed the expected increase in signal output to ~60,000 cps. These experiments confirmed that this CuAAC coupling reaction has the potential to selectively detect ligand-bound copper(I) in the presence of other biologically relevant metal ions.

## 3. Materials and Methods

### 3.1. General Procedure for the Synthesis of CuAAC Coupled Complexes ***14***–***21***

To Eu-DOTA complex **1a**, or Tb-DOTA complex **1b** (1 eq; dissolved at 20 mM concentration) in ^t^BuOH:H_2_O (2:1) was added TBTA (0.1 eq) and the mixture was allowed to stir for 15 min. Sodium ascorbate (0.2 eq; 0.1 M aq.) was added and the mixture was allowed to stir for 15 min followed by the addition of copper(II) sulfate (0.1 eq; 0.1 M aq.). After a further 15 min of stirring, the appropriate azide was added (1 eq) and the solution was allowed to stir under nitrogen at room temperature for 16 h. QuadraPure-IDA^®^ metal scavenger resin was added and the mixture was gently shaken at room temperature overnight, during which the blue colour of the solution faded. The resin was removed by filtration and the solvent was then removed *in vacuo* to give the crude triazole sensor.

### 3.2. Luminescence Measurements on Crude CuAAC Coupled Complexes ***14–21***

Excitation and emission fluorescence spectra were measured using a Horiba Jobin Yvon Fluoromax-P instrument. Lanthanide emission spectra were measured using a time-delayed setting on the same instrument.

Solutions of the crude mixtures of (**14**–**21**)-**Eu**/**Tb** were prepared (100 μM, H_2_O). The lanthanide luminescence intensity of each solution was measured at the stated λ_max_ of the antenna component (Table 1) using the following settings: time delay = 0.076 ms, slits = 10 nm, sample window = 5 ms, number of flashes = 20. The output was recorded as the relative emission (cps) at the wavelength (nm) corresponding to the most intense lanthanide transition (Table 1). A fold-response was calculated by comparing this emission (in cps) to that of a solution of the sensor components (100 μM each, 1:1 mixture in H_2_O) stirred at rt for 16 h, measured under identical conditions at the same wavelength.

### 3.3. Normalization of Output of CuAAC Coupled Complexes ***19*** and ***20*** by IR

IR were measured on a Perkin Elmer Paragon 100 FT-IR machine, and ASCII files of the resultant spectra used for subsequent data handling in Excel.

Samples were prepared as KBr die (at a final concentration of 0.5 mg azide, alkyne or crude complex in 120 mg KBr). The reproducibility of sample preparation was confirmed by preparing triplicate dies of pure sensor **19-Tb**. A spectrum of the 1:1 alkyne:azide starting component mixtures was generated from the reference spectra of the two starting materials using the principle of additivity of the two absorbers present. Normalization of absorbance between reference and crude product spectra was carried out at the wavenumber shown, allowing for a slight shift (<10 cm^−1^) in the C=O stretch between the two spectra (Table 2). Normalized luminescence output data (expressed as a fold increase) were calculated by estimating the % remaining azide in the normalized IR spectrum of the crude product mixture (Beer Lambert Law) and using this to determine the % reaction conversion by which the measured output could be proportionately adjusted.

### 3.4. Data for Purified Lead Complex ***19-Eu***

UV-vis (nm) λ_max_ = 219, 325; ^1^H-NMR δ (600 MHz, D_2_O) 32.76 (1H, s), 31.26 (1H, s), 30.60 (1H, s), 30.46 (1H, s), 7.67 (1H, s), 6.92-6.63 (3H, m), 5.08 (2H, s), 4.85 (2H, s), 3.82 (3H, s), 0.06 (1H, s), −0.37 (1H, s), −2.57 (1H, s), −2.67 (1H, s), −3.33 (1H, s), −4.55 (1H, s), −5.85 (1H, s), −7.18 (1H, s), −7.64 (1H, s), −7.95 (1H, s), −11.04 (1H, s), −11.33 (1H, s), −11.66 (1H, s), −12.22 (1H, s), −14.23 (1H, s), −14.40 (1H, s), −14.85 (1H, s), −15.67 (2H, s), −16.86 (1H, s); *m/z* (ESI+, H_2_O) 823 ([^153^EuM + H]^+^, 74), 821 ([^151^EuM + H]^+^, 44), 412 ([^153^EuM + 2H]^2+^, 100), 411 ([^151^EuM + 2H]^2+^, 80).

## 4. Conclusions

The detection of copper(I) species in a native biological setting (i.e., not one in which copper has been artificially introduced at non-physiological conditions) has the potential to enhance our understanding of a range of diseases, including those in which copper metabolism is misregulated (such as Menkes and Wilson’s diseases) and those in which copper is thought to be either causative or related to disease progression (such as Alzheimer’s disease). However, this detection is reliant on the design of sensors which are capable of reacting with predominantly ligand-bound copper, with a signal output which is not perturbed by the intrinsic properties of the biological milieu (e.g., inherent cellular fluorescence). For these reasons, sensors which are constructed using the exquisitely metal-ion selective CuAAC reaction [61] and which produce luminescent read-outs are particularly promising. By screening a range of potential antenna, using classes of fluorophore which are known to be cell-permeable, we have identified a sensor pairing which gives nearly two orders of magnitude signal increase over background, overcoming previous limitations in sensor design. Pairing this knowledge with recent advancements in ligand acceleration of the CuAAC reaction [62] could enable the very rapid detection of ligand-bound copper in an intracellular environment.

## Figures and Tables

**Table 1:** Sensor Output after CuAAC Reaction ^a^.

Entry	Alkyne	Azide	Sensor	λ_ex_/nm	Emission/cps ^b^ (wavelength/nm)	
1	**1a**	**6**	**14-Eu**	325	100	(615)
2	**1b**	**6**	**14-Tb**	325	240	(545)
3	**1a**	**7**	**15-Eu**	325	800	(615)
4	**1b**	**7**	**15-Tb**	325	338	(545)
5	**1a**	**8**	**16-Eu**	300	610	(615)
6	**1b**	**8**	**16-Tb**	300	845	(545) ^c^
7	**1a**	**9**	**17-Eu**	300	1300	(615)
8	**1b**	**9**	**17-Tb**	300	791	(545) ^c^
9	**1a**	**10**	**18-Eu**	345	5371	(593)
10	**1b**	**10**	**18-Tb**	345	6078	(545)
11	**1a**	**11**	**19-Eu**	325	56,592	(593)
12	**1b**	**11**	**19-Tb**	325	10,000	(545)
13	**1a**	**12**	**20-Eu**	345	16,665	(593)
14	**1b**	**12**	**20-Tb**	345	25,000	(545)
15	**1b**	**13**	**21-Tb**	350	4530	(545)

^a^ Reagents and Conditions: CuSO_4_ (10 mol%), NaAsc (20 mol%), TBTA (10 mol%), *^t^*BuOH:H_2_O (2:1), rt, 16 h; ^b^ Relative emission at specified wavelength (100 μM in H_2_O, time delay = 0.076 ms, slits = 10 nm, sample window = 5 ms, number of flashes = 20); ^c^ Decrease in signal from background.

**Table 2:** Normalized Values for Signal Increase for Sensors **19** and **20**.

Entry	Sensor	Output ^a^ (fold)	Wavenumber ^b^ (cm^−1^)	Conversion (%)	Normalized Output ^c^ (fold)
1	**19-Eu**	69	1612 (1612)	84	82
2	**19-Tb**	57	1614 (1615)	85	67
3	**20-Eu**	30	1628 (1624)	83	36
4	**20-Tb**	29	1614 (1626)	68	43

^a^ Signal increase over background; ^b^ Carbonyl bands used to normalize data for % conversion calculation: 1:1 component mixture (product spectra); ^c^ Sensor output normalized to 100% conversion for CuAAC reaction.

## Supplementary Materials

The following are available online: Schemes S1 and S2 and synthetic details for preparation of DOTA complexes **1a and 1b**, and azides **6**–**13** [63,64]; spectroscopic data for complexes **14**–**21**; Tables S1–S4: luminescence spectra for complexes **14**–**21**; Figure S1: ESI mass spectrum for crude reaction product **20-Tb**; Figure S2: HPLC chromatogram of crude reaction product **19-Tb**; Tables S5 and S6: normalized IR spectra for complexes **18** and **19**; ^1^H and ^13^C-NMR spectra for azides **7**, **8** and **9**; HPLC chromatogram, UV-vis, MS and ^1^H-NMR spectra for purified complex **19-Eu**.
