# Supplementary material for: Optimizing the Readout of Lanthanide-DOTA Complexes for the Detection of Ligand-Bound Copper(I)
Source: Molecules. 2017 May 14;22(5):802. doi: 10.3390/molecules22050802 (PMC6154328; doi:10.3390/molecules22050802)
Supplement: Supplementary file 1 [file molecules-22-00802-s001.pdf]

Supplementary Materials: Optimizing the Readout of Lanthanide-DOTA Complexes for the Detection of Ligand-Bound Copper(I)

Jill R. Hanna, Christopher Allan, Charlotte Lawrence, Odile Meyer, Neil D. Wilson and Alison N. Hulme

Contents

**S1**	**General Experimental**
**S2-S4**	**Synthesis of DOTA alkynes 1a and 1b**
**S4-S8**	**Synthesis of fluorescent azides 6-13**
**S8**	**General procedure for the CuAAC reaction**
**S9-S13**	**Spectroscopic data for sensors 14-21**
**S14-S17**	**Luminescence spectra for sensors 14-21**
**S18**	**Figure S1: ESI mass spectrum for crude reaction product 20-Tb**
**S18**	**Figure S2: HPLC chromatogram of crude reaction product 19-Tb**
**S19-S20**	**Normalized IR spectra for sensors 19 and 20**
**S21**	**^{1}H and ^{13}C NMR spectra for azides 7, 8 and 9**
**S24**	**LC, UV-vis, MS and ^{1}H NMR spectra for purified complex 19-Eu**
**S27**	**References**

General Experimental

All starting materials and reagents were purchased from commercial suppliers and were used as supplied. Anhydrous DCM was distilled from calcium hydride. Unless otherwise indicated, organic extracts were concentrated *in vacuo* using a rotary evaporator. Saturated aqueous solutions of inorganic salts are represented as (volume; sat. aq.). Flash column Chromatography was carried out on Merck Kieselger 60 (Merck 9385) under positive pressure by means of a hand pump. Eluent compositions are quoted as v/v ratios. Thin Layer Chromatography (TLC) was performed on MERCK 60F245 (0.25 mm) glass silica plates and visualised by ultraviolet (UV) light, potassium permanganate or ninhydrin stain.

^{1}H nuclear magnetic resonance (NMR) spectra were recorded at ambient temperature (unless otherwise stated) on Bruker AC250 (250 MHz), Bruker DPX360 (360 MHz), Bruker 500 (500 MHz) and Bruker 800 (800 MHz) Fourier Transform instruments. The data are presented as follows: chemical shift (in ppm on the scale relative to $\delta_{TMS} = 0$), multiplicity (s = singlet, d = doublet, t = triplet, q = quartet, m = multiplet), coupling constants and interpretation. ^{13}C nuclear magnetic resonance (NMR) spectra were recorded at ambient temperature (unless otherwise stated) on Bruker AC250 (62.9 MHz), Bruker DPX360 (90.6 MHz), Bruker 500 (201.3 MHz) and Bruker 800 (800 MHz). Fourier transform instruments were referenced to the solvent carbon peak. The data are presented as follows; chemical shift (in ppm on the δ scale), relative intensity and assignment. Where Distortionless Enhancement Polarisation Transfer (DEPT) spectra have been reported, the carbon signals due to methyl (CH_3), methylene (CH_2), methine (CH) and quaternary carbon (C) are assigned.

Infra-red spectra were recorded on a Perkin Elmer Paragon 100 FT-IR machine using 5 mm sodium chloride plates unless otherwise stated. The wavelengths of maximum absorbance (υ_{max}) are quoted in cm^{-1}. Melting points were determined on a Gallenkamp Electrothermal melting point apparatus and are uncorrected. Electronspray Ionisation (ESI) mass spectra were recorded on a Finnigan 450 or Micromass Platform instrument at the University of Edinburgh. Fast atom bombardment (FAB) mass spectra were obtained using a Kratos MS50TC mass spectrometer at The University of Edinburgh. Electrospray ionisation (ESI) mass

spectra were recorded on a Finnigan LCQ or Micromass Platform instrument at the University of Edinburgh. The parent ion or relevant fragment is quoted, followed by significant fragments and their percentages.

All HPLC samples were dissolved in solution (1:1, H_2O:MeOH) and filtered through 0.45 µm nylon syringe filters prior to analysis. HPLC samples were purified using a Waters 600E gradient pump and a Waters 486 tunable detector controlled by Waters Millenium software (version 3.2), which also processed the data. The reverse phase column used for analytical HPLC was a Luna 5µ C18(2) 100 Å (5 µm particle size, 250 mm × 4.6 mm i.d.) and samples were injected via a Rheodyne injector with a flow rate of 1 mL min^{-1}. The reverse phase column used for preparative HPLC was a Luna 5µ C18(2) 100 Å (5 µm particle size, 250 mm × 21.2 mm i.d.) and samples were injected via a Rheodyne injector with a flow rate of 5 mL min^{-1}. The solvent eluent system used was (A) = H_2O + 0.1% TFA; (B) = MeCN + 0.1% TFA. The gradient elution programme used was 5%→65% (B) over 70 min. Solvents were degassed on-line. Chromatographed peaks were monitored at 214 nm and the fractions collected manually.

Synthesis of DOTA alkynes 1a and 1b

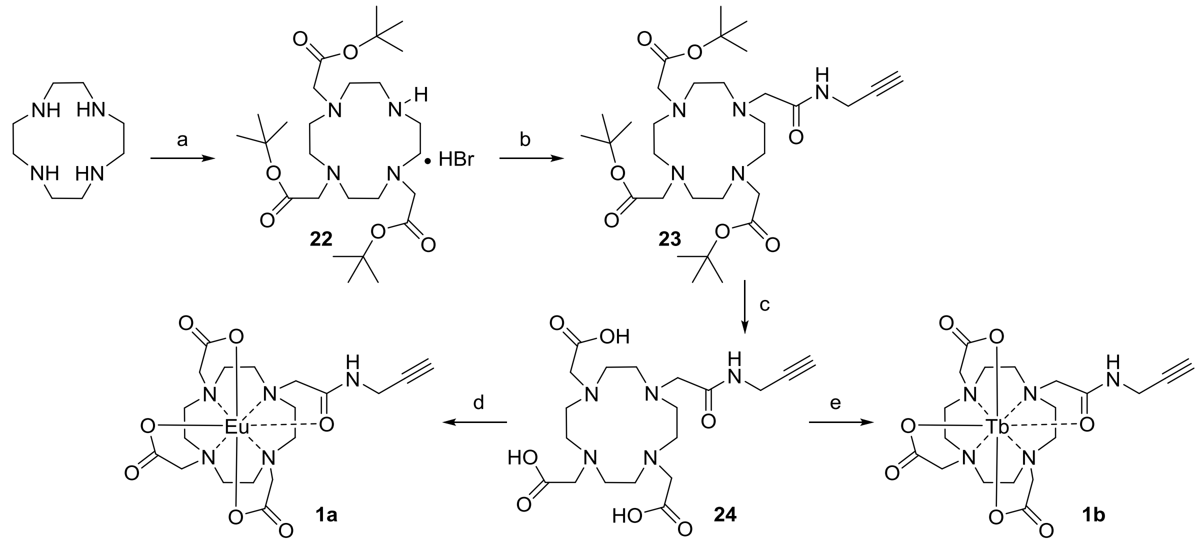

Scheme S1; Synthesis of lanthanide-DOTA complexes **1a** and **1b**. a) i) *tert*-butyl bromoacetate, NaOAc, DMAc 0 ˚C→ rt, 7 d, ii) KBr, H_2O, $NaHCO_3$, pH 9, 68% [25]; b) $ClCH_2CONHCH_2C{\equiv}CH$, K_2CO_3, MeCN, 48 h, 90%; c) TFA, rt, 16 h, quant. [11]; d) $Eu(OTf)_3$, H_2O, pH 6, 60 °C, 4 h, quant. [11]; e) $Tb(OTf)_3$, H_2O, pH 6, 60 °C, 4 h, 95%.

Tri-*tert*-butyl 1,4,7,10-tetraazacyclododecane-4,7,10-triacetate•HBr 22

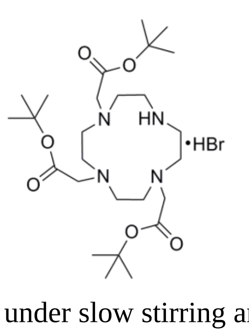

tert-Butylbromoacetate (7.71 ml, 52.2 mmol) in *N,N*-dimethylacetamide (15 ml) was added dropwise to a stirred suspension of 1,4,7,10-tetraazacyclododecane (3.00 g, 17.4 mmol) and sodium acetate (4.29 g, 52.2 mmol) in *N,N*-dimethylacetamide (40 ml) at 0 ℃ over 25 min. After the last addition the reaction was allowed to warm to room temperature. The reaction vessel was put under an atmosphere of nitrogen and the white suspension was stirred for 7 days. The reaction was poured into warm water (200 ml, 50 ℃), containing dissolved KBr (3.00 g, 25.2 mmol), to give a clear yellow solution. The pH was adjusted to 9 by the addition of solid $NaHCO_3$ and a white crystalline material precipitated out. The suspension was allowed to cool to room temperature under slow stirring and then the precipitate was allowed to sediment without stirring for 4 h. The precipitate was removed by filtration and dried *in vacuo*. The solid was purified by column chromatography (DCM:MeOH, 90:10) to give triacetate **22** as a pale cream solid (2.03 g, 68%). $\mathbf{R_f}$ (DCM:MeOH, 90:10) = 0.8; **IR** 3436 (NH), 1729 (C=O); **mp** 181 – 183 ℃; **^{1}H NMR** δ (400 MHz, DMSO-d_6) 8.84 (2H, s, NH_2), 3.41 (2H, s, CH_2), 3.33 (4H, d, J = 11.3 Hz, 2 × CH_2), 2.97 (4H, br s, 2 × CH_2), 2.84 (4H, br s, 2 × CH_2), 2.69 (8H, br d, J = 7.9 Hz, 4 × CH_2), 1.42 (27H, d, J = 3.4 Hz, C(CH_3)$_3$); **^{13}C NMR** δ (63 MHz, DMSO-d_6) 170.5 (*C*=O), 170.0 (*C*=O), 80.6 (*C*(CH$_3$)$_3$), 80.5 (*C*(CH$_3$)$_3$), 56.0 (*C*H$_2$), 51.9 (*C*H$_2$), 49.7 (*C*H$_2$), 48.4 (*C*H$_2$), 45.6 (*C*H$_2$), 27.9 (*C*H$_3$); ***m/z*** (ESI+, MeOH) 515 ([M+H]$^+$, 100).

^{1}H and ^{13}C NMR spectroscopic data in good agreement with the literature [25].

2-Chloro-*N*-prop-2-ynyl-acetamide

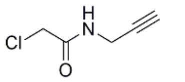

A solution of triethylamine (1.20 ml, 9.00 mmol) and propargylamine (0.600 ml, 9.00 mmol) in anhydrous THF (20 ml) were added dropwise to a stirred solution of chloroacetylchloride (0.700 ml, 9.00 mmol) in anhydrous THF (30 ml) at -78 ℃ over 1 h. The mixture was stirred for 3.5 h at -78 ℃, allowed to warm to room temperature and stirred at this temperature for 1 h. The solution was filtered and the solvent removed *in vacuo*. The resulting solid was purified by column chromatography (EtOAc:Cyclohexane, 2:1) to yield 2-chloro-*N*-prop-2-ynyl-acetamide as a yellow solid (1.52 g, 75%). $\mathbf{R_f}$ (EtOAc:Cyclohexane, 2:1) = 0.3; **mp** 65 – 67 ℃, lit.[26] 67 – 68 ℃; **IR** 3343 (NH), 2098 (C≡C), 1652 (C=O); **^{1}H NMR** δ (250 MHz, $CDCl_3$) 6.90 (1H, br s, N*H),* 4.11 (2H, dd, *J* = 5.4, 2.6 Hz, C*H*$_2$NH), 4.08 (2H, s, C*H*$_2$Cl), 2.30 (1H, t, *J* = 2.6 Hz, C*H*); **^{13}C NMR** δ (63 MHz, $CDCl_3$) 165.6 (*C*=O), 78.9 (*C*≡CH), 72.0 (C≡*C*H), 42.2 (*C*H$_2$NH), 29.4 (*C*H$_2$Cl); ***m/z*** (ESI+, MeOH) 132 ($[M+H]^+$, 100).
^{1}H and ^{13}C NMR spectroscopic data in good agreement with the literature [26].

(4,7-Bis-*tert*-butoxycarbonylmethyl-10-prop-2-ynylcarbamoylmethyl-1,4,7,10 tetraaza-cyclododec-1-yl)-acetic acid *tert*-butyl ester 23

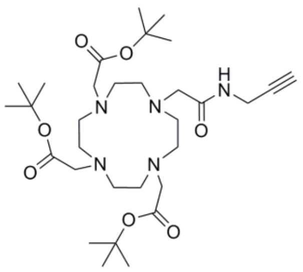

A solution of amine **25** (1.03 g, 1.99 mmol), 2-chloro-*N*-prop-2-ynyl-acetamide (0.262 g, 1.99 mmol) and K_2CO_3 (0.550 g, 3.98 mmol) in acetonitrile (200 ml) was heated to reflux under nitrogen for 48 h. After removal of the solvent *in vacuo*, the residual mixture was dissolved in dichloromethane (50 mL), filtered and evaporated to yield alkyne **23** as a golden oil (0.99 g, 90%) which was used without further purification for subsequent reactions. $\mathbf{R_f}$ (DCM:MeOH, 90:10) = 0.8; **IR** 3413 (NH), 1724 (C=O), 1668 (C=O); **^{1}H NMR** δ (250 MHz, $CDCl_3$) 9.23 (1H, br s, CON*H*CH$_2$), 3.98 (1H, dd, *J* = 5.7, 2.4 Hz, CH$_2$C≡C*H*), 3.38 (s, 8H, CH$_2$), 2.88-2.79 (2H, m, N*H*), 2.76 (16H, s, 8 × C*H*$_2$), 2.66-2.56 (2H, m, N*H*), 1.40 (27H, br s, 9 × C*H*$_3$); **^{13}C NMR** δ (63 MHz, $CDCl_3$) 170.5 (*C*O), 170.1 (*C*O), 81.4 (*C*(CH$_3$)$_3$), 80.2 (*C*≡CH), 56.9 (C≡*C*H), 52.8 (N*C*H$_2$*C*O) 51.4 (N*C*H$_2$CO), 50.3 (*C*H$_2$), 47.0 (*C*H$_2$), 27.0 (3 × *C*H$_3$); ***m/z*** (ESI+, MeOH) 648 ($[M+K]^+$, 10). 632 ($[M+Na]^+$, 70%), 610 ($[M+H]^+$, 100).
^{1}H and ^{13}C NMR spectroscopic data in good agreement with the literature [11].

(4,7-Bis-carboxymethyl-10-prop-2-ynylcarbamoylmethyl-1,4,7,10 tetraaza-cyclododec-1-yl)-acetic acid 24 (TFA Salt)

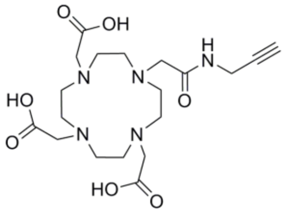

Tri-*tert*-butyl ester **26** (0.25 g, 0.40 mmol) was stirred in trifluoroacetic acid (20 ml) for 24 h at room temperature. The solvent was removed *in vacuo* and the resulting residue was dissolved in the minimum amount of methanol. Diethyl ether was added (50 ml), resulting in the precipitation of a cream/brown solid which was filtered and air dried to give the TFA salt of DOTA-alkyne **24** as a cream/brown solid (0.18 g, >99%). **IR** 3283 (OH/NH), 1700 (C=O), 1684 (C=O); **mp** 110 ℃ (decomp); **^{1}H NMR** δ (500 MHz, D_2O, pH 1) 3.92 (2H, br s, C*H*$_2$C≡CH), 3.83 (4H, br s, 2 × C*H*$_2$), 3.63 (4H, br s, 2 × C*H*$_2$), 3.37 (8H, br s, 4 × C*H*$_2$), 3.19 (8H, br s, 4 × C*H*$_2$), 2.58 (1H, t, C≡C*H*); **^{1}H NMR** δ (500 MHz, D_2O, pH 14) 3.92 (2H, br s, C*H*$_2$C≡CH), 3.83 (2H, br s, C*H*$_2$), 3.03 (6H, br s, 3 × C*H*$_2$), 2.69 - 2.64 (8H, br m, 4 × C*H*$_2$), 2.37 (8H, br s, 4 × C*H*$_2$), 2.18 (1H, m, C≡C*H*); **^{13}C NMR** δ (126 MHz, D_2O, pH 1) 175.1 (2C), 170.1 (C), 80.1 (C), 72.6 (CH), 66.7 (2 × C*H*$_2$), 55.7 (CH$_2$), 53.7 (CH$_2$), 52.4 (CH$_2$), 49.7 (CH$_2$), 48.6 (CH$_2$), 42.9 (CH$_2$), 29.4 (2 × C*H*$_2$); ***m/z*** (ESI+) 480 ($[M+K]^+$, 100%), 464 ($[M+Na]^+$, 40), 442 ($[M+H]^+$, 35).
^{1}H and ^{13}C NMR spectroscopic data in good agreement with the literature [11].

General Procedure 1: Preparation of Lanthanide complexes

A solution of the appropriate deprotected DOTA compound **24** (1.0 eq) was prepared in water and adjusted to pH 7 by addition of KOH (0.1 M aq.). The appropriate lanthanide (LnX_3, 1.0 eq) was dissolved in water and added to the DOTA solution. After mixing thoroughly for 15 min, the solution was re-adjusted to pH 6 using KOH (0.1 M aq.) and the reaction mixture was stirred at 60 ℃ for 4 h. The solution was once again adjusted to pH 6 and the solvent was removed *in vacuo.* The colourless solid was dissolved in EtOH, the insoluble salts removed by filtration and the solvent was removed *in vacuo* to yield the appropriate lanthanide-DOTA complex.

Europium(III)-DOTA complex 1a

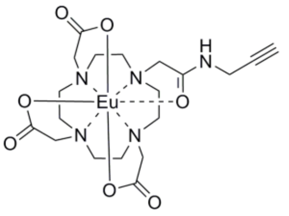

According to **general procedure 1** DOTA-alkyne **24** (0.18 g, 0.41 mmol) and europium triflate (0.24 g, 0.41 mmol) in water (4 ml) afforded Eu-DOTA-complex **1a** as a colourless solid (0.25 g, >99%). **mp** 197 – 200 ℃; **IR** (KBr disk) 3430 (OH/NH), 1683 (C=O), 1594 (C=O); **^{1}H NMR** δ (400 MHz, D_2O) 33.07 (s), 31.70 (s), 30.81 (s), 30.46 (s), 22.06 (s), 20.11 (s), 13.37 – 5.10 (m), 6.12 (s), 4.81 (s), 4.27 – 1.5 (m), 1.24 (s), 1.14 – 1.10 (m), 0.08 (s), -0.33 (s), -2.37 (s), -3.42 (s), -4.26 (s), -5.69 (s), -7.14 (s), -7.72 (s), -8.08 (s), -8.97 (s), -10.91 (s), -11.63 (s), -12.37 (s), -13.94 – 15.13 (m), -15.66 (s), -16.69 (s); ***m/z*** (ESI+, MeOH) 630 ([^{151}EuM+K]$^+$, 10%), 628 ([^{153}EuM+K]$^+$, 8), 592 ([^{153}EuM+H]$^+$, 20), 590 ([^{151}EuM+H]$^+$, 18), 269 (30), 226 (50), 209 (100).
^{1}H NMR spectroscopic data in good agreement with the literature [11].

Terbium(III)-DOTA complex 1b

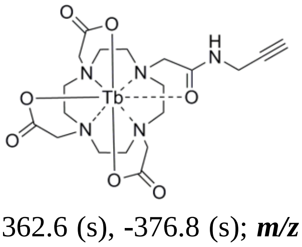

According to **general procedure 1**, DOTA-alkyne **24** (0.55 g, 1.24 mmol), terbium triflate (0.75 g, 1.24 mmol) and water (10 ml) afforded Tb-DOTA complex **1b** as a colourless solid. (0.70 g, 95%). **mp** 198 ℃; **IR** (KBr disc) 3423 (OH/NH), 3284 (OH/NH), 2100 (C≡C), 1684 (C=O), 1620 (C=O); **^{1}H NMR** δ (600 MHz, D_2O) 255.9 (s), 243.1 (s), 237.1 (s), 212.7 (s), 199.4 (s), 115.9 (s), 109.0 (s), 60.1 (s), 47.0 – 43.4 (m), 19.5 (s), 11.2 (s), 3.7 (s), 2.0 (s), 1.4 – 1.2 (m), -0.7 (s), -2.0 (s), -63.3 (s), -75.2 (s), -103.1 (s), -113.8 (s), -116.8 (s), -127.3 (s), -362.6 (s), -376.8 (s); ***m/z*** (ESI+, MeOH) 636 ([M+K]$^+$, 13%), 620 ([M+Na]$^+$, 11), 615 (15), 598 ([M+H]$^+$, 100), 480 (15); **HRMS** (ESI-, MeOH) [M-H]$^-$ found 596.1159, $C_{19}H_{27}N_5O_7Tb$ requires 596.1169.

Synthesis of fluorescent azides 6-13

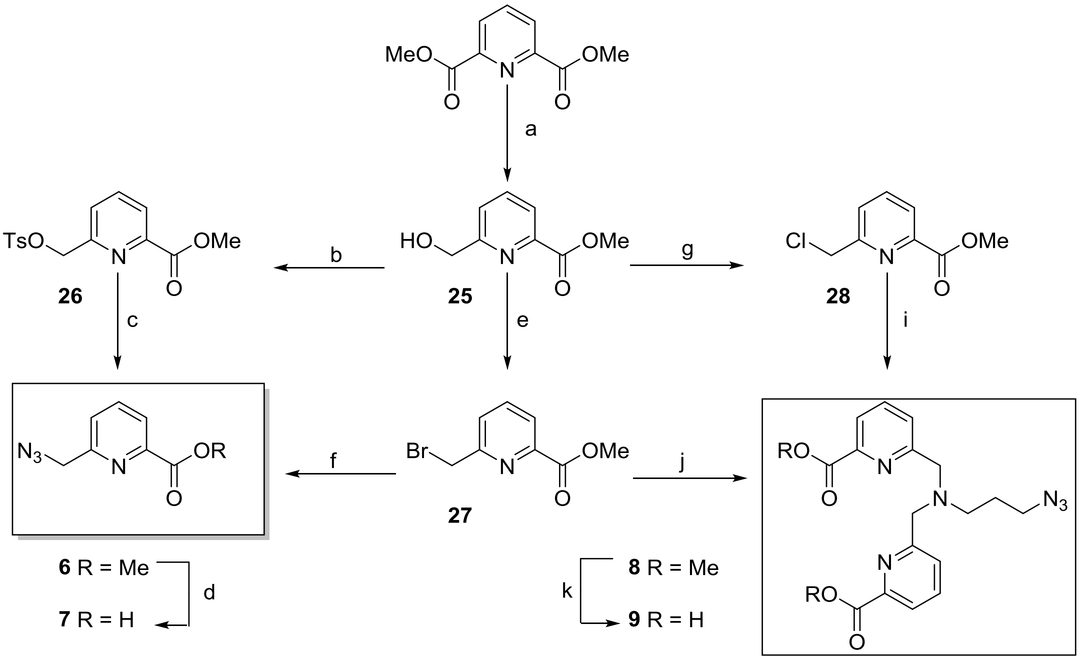

Scheme S2; a) $NaBH_4$, MeOH, rt, 3 h, 68%; b) TsCl, toluene, NEt_3, 72 h, rt, 62%; c) NaN_3, H_2O, toluene, TBAB, 80 °C, 18 h, 55%; d) LiOH•H_2O, H_2O, 16 h, rt, ion exchange resin, 54%; e) PPh_3, Br_2, DCM, 0 °C, 15 min, rt, 3 h, 70%; f) NaN_3, H_2O, toluene, TBAB, 80°C, 18 h, 58%; g) $SOCl_2$, 0°C, 1 h, 88%; h) H_2O, NaN_3, 15 h, 80 °C, assumed quant; i) $H_2NCH_2CH_2CH_2N_3$, K_2CO_3, MeCN, 80 °C, 20 h, 45%; j) $H_2NCH_2CH_2CH_2N_3$, K_2CO_3, MeCN, 80 °C, 20 h, 41%; k) HCl (6 M aq), 95°C, 2 h, 81%.

6-Hydroxymethyl-pyridine-2-carboxylic acid methyl ester 25

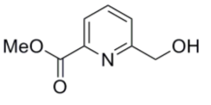

A solution of pyridine-2,6-dicarboxylic acid dimethyl ester (2.00 g, 10.2 mmol) in methanol (100 ml) was cooled to 0 ℃. Sodium borohydride (0.78 g, 20.5 mmol) was added portion-wise over 40 min. The reaction mixture was stirred for 3 h at room temperature and then neutralised with an aqueous saturated NH_4Cl solution. After extraction with DCM (3 ×

500 mL), the combined organic layers were dried with Na_2SO_4, filtered and the solvent was removed *in vacuo*. The crude product was purified by column chromatography (hexane:EtOAc, 1:1, then 1:2) to give mono- alcohol **25** as a colourless solid (1.17 g, 68%). **R$_f$** (Hexane:EtOAc, 1:1) = 0.2; **mp** 87 – 88 °C, lit.[63] 88 °C; **IR** 3366 (OH), 1729 (C=O); **^{1}H NMR** δ (250 MHz, $CDCl_3$) 7.99 (1H, d, *J* = 7.7 Hz, Ar*H*), 7.82 (1H, t, *J* = 7.7 Hz, Ar*H*), 7.55 (1H, d, *J* = 7.8 Hz, Ar*H*), 4.85 (2H, s, C*H*$_2$), 3.95 (3H, s, C*H*$_3$); **^{13}C NMR** δ (63 MHz, $CDCl_3$) 165.6 (C), 160.5 (C), 146.9 (C), 137.8 (CH), 124.1 (CH), 123.8 (CH), 64.7 (CH_2), 52.9 (CH_3); ***m/z*** (ESI+) 190 ($[M+Na]^+$, 100%).
^{1}H and ^{13}C NMR spectroscopic data in good agreement with the literature [36].

6-(Toluene-4-sulfonyloxymethyl)-pyridine-2-carboxylic acid methyl ester 26

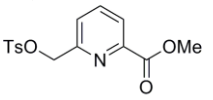

A solution of mono-alcohol **25** (1.10 g, 5.79 mmol) in dry toluene (50 ml) was cooled to 0 °C and *p*-toluenesulfonyl chloride (1.99 g, 10.5 mmol) and triethylamine (1.50 ml, 10.5 mmol) were added. The reaction mixture was warmed to room temperature and stirred for 18 h. The solvent was removed from the reaction mixture *in vacuo*. The crude product was purified by chromatography (Hexane:EtOAc, 7:3) to give tosyl methyl ester **26** as a pale brown solid. (1.15 g, 62%). **R$_f$** (Hexane:EtOAc, 7:3) = 0.2; **mp** 87 – 88 °C; **IR** 1725 (C=O), 1176 (S=O); **^{1}H NMR** δ (250 MHz, $CDCl_3$) 8.05 (1H, d, *J* = 7.7 Hz, Ar*H*), 7.86 (3H, m, Ar*H*), 7.67 (1H, d, *J* = 7.9 Hz, Ar*H*), 7.34 (2H, d, *J* = 8.4 Hz, Ar*H*), 5.22 (2H, s, C*H*$_2$), 3.98 (3H, s, OC*H*$_3$), 2.44 (3H, s, TsC*H*$_3$); **^{13}C NMR** δ (63 MHz, $CDCl_3$) 165.3 (*C*), 154.8 (*C*), 147.6 (*C*), 145.4 (*C*), 138.3 (*C*H), 132.5 (*C*), 130.1 (2 × *C*H), 128.2 (2 × *C*H), 124.9 (*C*H), 124.8 (*C*H), 71.4 (*C*H$_2$), 53.2 (*C*H$_3$), 21.8 (*C*H$_3$); ***m/z*** (ESI+) 344 ($[M+Na]^+$, 100%), 322 ($[M+H]^+$, 15).
^{1}H spectroscopic data in good agreement with the literature [64].

6-Bromomethyl-pyridine-2-carboxylic acid methyl ester 27

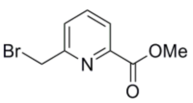

Triphenylphosphine (0.76 g, 2.90 mmol) was added to a solution of bromine (1.78 ml, 4.29 mmol) in anhydrous DCM (25 ml) at 0 °C and the solution was stirred for 15 min. The resulting slurry was added dropwise to mono-alcohol **25** (0.28 g, 1.47 mmol) and allowed to warm to room temperature. The reaction was monitored by tlc and mass spectrometry. After 3 h the solution was quenched with $Na_2S_2CO_3$ solution (10 ml, 0.1 M aq.) and water (10 ml) and the aqueous solution was extracted with DCM (3 × 30 ml). The organic layer was dried with Na_2SO_4 and the solvent was removed *in vacuo.* The crude product was purified using column chromatography (Hexane:EtOAc, 1:1) to yield bromide **27** as a colourless solid (0.72 g, 70%); **R$_f$** (EtOAc:Hexane, 1:1) = 0.6; **mp** 68 – 70 °C; **IR** 1739 (C=O); **^{1}H NMR** δ (500 MHz, $CDCl_3$) 8.05 (1H, dd, *J* = 7.7, 1.1 Hz, Ar*H*), 7.85 (1H, t, *J* = 7.8 Hz, Ar*H*), 7.68 (1H, dd, *J* = 7.8, 1.0 Hz, Ar*H*), 4.63 (2H, s, C*H*$_2$), 4.00 (3H, s, C*H*$_3$); **^{13}C NMR** δ (126 MHz, $CDCl_3$) 165.3 (*C*=O), 157.3 (*C*), 147.5 (*C*), 138.1 (*C*H), 127.0 (*C*H), 124.4 (*C*H), 53.0 (*C*H$_3$), 33.2 (*C*H$_2$Br); ***m/z*** (EI, MeOH) 231 ($[^{81}BrM+H]^+$, 4%), 229 ($[^{79}BrM]^+$, 4), 201 (28), 199 (29), 173 (97), 171 (100), 150 (13), 91 (35).
^{1}H and ^{13}C NMR spectroscopic data in good agreement with the literature [63].

6-Azidomethyl-pyridine-2-carboxylic acid methyl ester 6

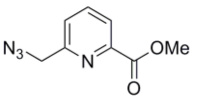

Method A: Tosyl methyl ester **26** (0.430 g, 1.34 mmol), sodium azide (0.700 g, 10.7 mmol), tetrabutylammonium bromide (0.04 g, 0.134 mmol), water (10 ml) and toluene (10 ml) were heated to 80 °C for 18 h. The reaction mixture was cooled to room temperature and extracted with DCM (3 × 20 ml). The combined organics were dried with $NaSO_4$, filtered and the solvent was removed *in vacuo*. The crude product was purified by chromatography (Hexane:EtOAc, 7:3) to give azide methyl ester **6** as a colourless oil. (0.14 g, 55%).
Method B: Bromide **27** (0.25 g, 1.1 mmol), sodium azide (0.58 g, 8.9 mmol), tetrabutylammonium bromide (0.033 g, 0.11 mmol), water (8 ml) and toluene (8 ml) were heated to 80 °C for 18 h. The reaction mixture was cooled to room temperature and extracted with DCM (3 × 15 ml). The combined organics were dried with $NaSO_4$, filtered and the solvent was removed *in vacuo*. The crude product was purified by chromatography (Hexane:EtOAc, 7:3) to give azide methyl ester **6** as a colourless oil. (0.12 g, 58%). **R$_f$** (EtOAc) = 0.6; **IR** 2106 (N_3), 1725 (C=O); **^{1}H NMR** δ (250 MHz, $CDCl_3$) 8.12 – 8.03 (1H, m, Ar*H*), 7.88 (1H, t, *J* = 7.8 Hz, Ar*H*), 7.63 – 7.53 (1H, m, Ar*H*), 4.62 (2H, s, C*H*$_2$), 3.99 (3H, s, C*H*$_3$); **^{13}C NMR** δ (63 MHz, $CDCl_3$) 165.5 (*C*), 156.5 (*C*), 147.9 (*C*), 138.2 (*C*H), 125.5 (*C*H), 124.4 (*C*H), 55.6 (*C*H$_2$), 53.1 (*C*H$_3$); ***m/z*** (ESI+) 215 ($[M+Na]^+$, 94%) , 193 ($[M+H]^+$, 28).
^{1}H and ^{13}C NMR spectroscopic data in good agreement with the literature [36].

6-Azidomethyl-pyridine-2-carboxylic acid 7

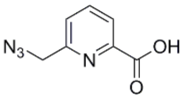

A solution of lithium hydroxide monohydrate (27.9 mg, 0.667 mmol) in water (2 ml) was slowly added to a solution of azide methyl ester **6** (32.2 mg, 0.167 mmol) and methanol (2 ml) and stirred for 15 min. The methanol was removed from the reaction mixture *in vacuo* and ion exchange resin (0.20 g) was added to the aqueous solution. The reaction mixture was stirred until pH 3 was obtained, whereupon the resin was removed by filtration. The water was removed using a freeze drier to yield carboxylic acid **7** as a colourless solid (16.0 mg, 54%). **mp** 240 ℃ (decomp); **IR** (KBr disc) 3388 (OH), 2108 (N_3), 1618 (C=O); **^{1}H NMR** δ (500 MHz, D_2O) 7.93 (1H, t, *J* = 7.7 Hz, ArH), 7.82 (1H, d, *J* = 7.7 Hz, ArH), 7.54 (1H, d, *J* = 7.7 Hz, ArH), 4.56 (2H, s, CH_2); **^{13}C NMR** δ (126 MHz, D_2O) 172.9 (C), 154.7 (C), 153.5 (C), 139.0 (CH), 124.3 (CH), 122.8 (CH) 54.7 (CH_2); ***m/z*** (ESI-) 177 ($[M-H]^-$, 100%), 62 (54).

Hazards with the handling of azides and sodium azide:
CAUTION, unstable. Avoid; heat, sources of ignition, moisture, shock and friction. Incompatible with strong oxidising agents, mineral acids, water, halogen acids and halogen compounds, barium carbonate, bromine, CS_2, mercury, dimethyl sulphate, common metals, especially brass, copper, lead, silver, strong acids. Poison and harmful by inhalation, ingestion, or by skin contact. Material absorbed through the skin and toxic to the environment. For a review covering the synthesis and reactivity of azides, see: Bräse, S.; Gil, C.; Knepper, K.; Zimmermann, V. *Angew. Chem. Int. Ed.*, **2005**, *44*, 5188 - 5248.

6-Chloromethyl-pyridine-2-carboxylic acid methyl ester 28

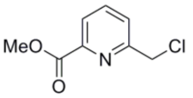

Mono-alcohol **25** (0.35 g, 1.84 mmol) was cooled to 0 ℃ and thionyl chloride (1.09 ml, 14.7 mmol) was added dropwise. The reaction mixture was stirred for 1.5 h at 0 ℃ and monitored by tlc. The reaction mixture was warmed to room temperature and the solvent was removed *in vacuo*. Toluene (15 ml) was added to the residue and the solution was washed with sat $NaHCO_3$ (2 × 5 ml). The combined organics were dried with $NaSO_4$, filtered and the solvent was removed *in vacuo*. The crude product was purified by chromatography (EtOAc) to give chloride **28** as a pale yellow solid (0.30 g, 88%). **R_f** (EtOAc) = 0.9; **mp** 50 – 52 ℃; **IR** 1748 (C=O); **^{1}H NMR** δ (250 MHz, $CDCl_3$) 8.07 (1H, d, *J* = 7.7, Ar*H*), 7.89 (1H, t, *J* = 7.8, Ar*H*), 7.72 (1H, d, *J* = 7.8, Ar*H*), 4.76 (2H, s, C*H*$_2$), 4.00 (3H, s, C*H*$_3$); **^{13}C NMR** δ (63 MHz, $CDCl_3$) 165.5 (*C*), 157.3 (*C*), 147.6 (*C*), 138.3 (*C*H), 126.3 (*C*H), 124.6 (*C*H), 53.2 (*C*H$_3$), 46.3 (*C*H$_2$); ***m/z*** (ESI+) 208 ($[M+Na]^+$, 100%); **HRMS** (ESI+, MeOH) $[M+H]^+$ $C_8H_9ClNO_2$ requires 186.0316, found 186.0316.
^{1}H and ^{13}C NMR spectroscopic data in good agreement with the literature [37].

3-azidopropan-1-amine

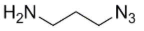

To a solution of 3-chloropropan-1-amine hydrochloride salt (1.00 g, 7.69 mmol) in water (4 ml) was added NaN_3 (1.49 g, 22.7 mmol) and the reaction was heated at 80 ℃ for 15 h. The solution was basified with KOH (1.10 g, 19.2 mmol) and extracted with diethyl ether (3 × 5 ml). The combined organics were dried over anhydrous Na_2SO_4, filtered and the solvent was removed (almost to dryness) *in vacuo* to give 3-azidopropan-1-amine as a colourless oil (0.761 g, quant.). **IR** 3415 (NH), 2101 (N_3); **^{1}H NMR** δ (250 MHz, $CDCl_3$) 3.16 (2H, t, *J* = 6.7 Hz, C*H*$_2$), 2.59 (2H, t, *J* = 6.8 Hz, C*H*$_2$), 1.51 (2H, qn, *J* = 6.8 Hz, C*H*$_2$); **^{13}C NMR** δ (63 MHz, $CDCl_3$) 48.6 (*C*H$_2$), 38.8 (*C*H$_2$), 32.0 (*C*H$_2$); ***m/z*** (ESI+, MeOH) 101 ($[M+H]^+$, 35%).

6-{[(3-Azido-propyl)-(6-methoxycarbonyl-pyridin-2-ylmethyl)-amino]-methyl}-4-methyl-pyridine-2-carboxylic acid methyl ester 8

Method A: To a solution of chloride **28** (0.389 g, 2.09 mmol) in anhydrous acetonitrile (3.5 ml), freshly

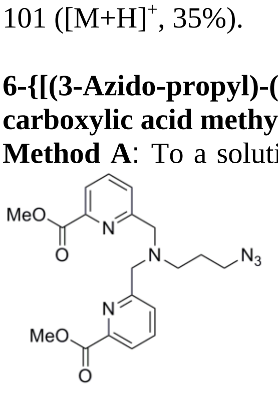

prepared 3-azidopropan-1-amine (0.104 g, 1.04 mmol) and anhydrous K_2CO_3 (0.721 g, 5.22 mmol) were added. The reaction mixture was heated to reflux for 30 h. The solution was filtered to remove the inorganic salts and washed with sat. $NaHCO_3$ (3 × 10 ml). The combined organics were dried with Na_2SO_4, filtered and the solvent removed *in vacuo*. The crude residue was purified by column chromatography (Cyclohexane:EtOAc, 3:1) to give dimethyl ester **8** as a colourless oil (0.375 g, 45%).

Method B: To a solution of bromide **27** (0.194 g, 0.842 mmol) in anhydrous acetonitrile (2 ml), freshly prepared 3-azidopropan-1-amine (0.104 g, 0.104 mmol) and anhydrous K_2CO_3 (0.290 g, 2.10 mmol) were added. The reaction mixture was heated to reflux for 30 h. The solution was filtered to remove the inorganic salts and washed with sat. $NaHCO_3$ (3 × 10 ml). The combined organics were dried with Na_2SO_4, filtered

and the solvent removed *in vacuo*. The crude residue was purified by column chromatography (Cyclohexane:EtOAc, 3:1) to give dimethyl ester **8** as a colourless oil (0.137 g, 41%). **R_f** (Cyclohexane:EtOAc, 3:1) = 0.3; **IR** 2096 (N_3), 1724 (CO); **^{1}H NMR** δ (250 MHz, $CDCl_3$) 8.00 (2H, dd, *J* = 7.4, 1.3 Hz, Ar*H*), 7.82 (2H, t, *J* = 7.6 Hz, Ar*H*), 7.74 (2H, dd, *J* = 7.8, 1.3 Hz, Ar*H*), 3.99 (6H, s, 2 × OCH_3), 3.93 (4H, s, 2 × ArCH_2N), 3.31 (2H, t, *J* = 6.7 Hz, CH_2N_3), 2.66 (2H, t, *J* = 6.9 Hz, CH_2N), 1.80 (2H, qn, *J* = 6.8 Hz, $CH_2CH_2CH_2N_3$); **^{13}C NMR** δ (63 MHz, $CDCl_3$) 165.9 (2C, *C*O), 160.3 (2C, *C*), 147.6 (2C, *C*), 137.6 (2C, *C*H), 126.1 (2C, *C*H), 123.9 (2C, *C*H), 60.4 (2C, *C*H_2), 53.1 (2C, *C*H_3), 51.6 (*C*H_2), 49.4 (*C*H_2), 26.7 (*C*H_2); ***m/z*** (ESI+, MeOH) 421 ($[M+Na]^+$, 85%), 399 ($[M+H]^+$, 100); **HRMS** (ESI+, MeOH) $[M+H]^+$ $C_{19}H_{23}N_6O_4$ requires 399.1775, found 399.1777.

6-{[(3-Azido-propyl)-(6-carboxy-pyridin-2-ylmethyl)-amino]-methyl}-4-methyl-pyridine-2-carboxylic acid 9

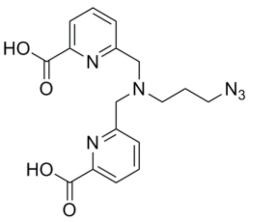

A solution of dimethyl ester **8** (0.0578 g, 0.145 mmol) in hydrochloric acid (6 ml, 6 M aq.) was heated at 95 °C for 2 h. The reaction mixture was concentrated in *vacuo* and the crude residue was freeze-dried to yield dicarboxylic acid **9** as a yellow solid (0.0435 g, 81%). **mp** 194 – 195 °C (decomp); **IR** (KBr disk) 3465 (OH), 2108 (N_3), 1647 (C=O); **^{1}H NMR** δ (500 MHz, D_2O) 7.79 (2H, d, *J* = 7.6, Ar*H*), 7.72 (2H, t, *J* = 7.8 Hz, Ar*H*), 7.38 (2H, d, *J* = 7.6 Hz, Ar*H*), 4.55 (4H, s, 2 × ArCH_2N), 3.45 – 3.40 (2H, m, CH_2N_3), 3.34 (2H, t, *J* = 6.3 Hz, CH_2N), 2.07 – 1.98 (2H, m, $CH_2CH_2CH_2N_3$); **^{13}C NMR** δ (63 MHz, D_2O) 167.4 (2C, *C*) 150.2 (2C, *C*), 147.0 (2C, *C*), 140.2 (2C, *C*H), 128.7 (2C, *C*H), 125.7 (2C, *C*H), 58.8 (*C*H_2), 55.0 (*C*H_2), 48.5 (2C, *C*H_2), 23.6 (*C*H_2); ***m/z*** (ESI+, MeOH) 371 ($[M+H]^+$, 100%); **HRMS** (ESI+, MeOH) $[M+H]^+$ $C_{17}H_{19}N_6O_4$ requires 371.1462, found 371.1464.

7-Azido-4-methyl-chromen-2-one 10

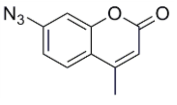

7-Amino-4-methylcoumarin (50.0 mg, 0.285 mmol) was dissolved in a solution of concentrated sulfuric acid (0.06 ml) and water (0.17 ml). The resultant solution was cooled to 0 °C, and a solution of sodium nitrite (35.0 mg, 0.507 mmol) in water (0.2 ml) was added dropwise with stirring. The solution was stirred at 0 °C for a further 15 min, whereupon a solution of sodium azide (50.0 mg, 0.769 mmol) in water (0.1 ml) was added with vigorous stirring. The mixture was stirred for 1 h at 0 °C, then overnight at room temperature. The reaction mixture was basified with sat. Na_2CO_3 solution and extracted with DCM (3 × 1 ml). The combined organics were washed with water, dried over $MgSO_4$, filtered, and the solvent was removed *in vacuo*. The crude solid was purified by column chromatography (DCM) to yield azide **10** as an orange solid (42.1 mg, 72%). **R_f** (DCM) = 0.4; **mp** 115 – 116 °C; **IR** 2094 (N_3), 2119 (N_3) 1722 (C=O), 1608 (C=C); **^{1}H NMR** δ (250 MHz, $CDCl_3$) 7.55 (1H, dd, *J* = 7.5, 1.7 Hz, Ar*H*), 6.96 – 6.92 (2H, m, Ar*H*), 6.20 (1H, br s, Ar*H*), 2.40 (3H, d, *J* = 1.1 Hz, CH_3); **^{13}C NMR** δ (63 MHz, $CDCl_3$) 160.4 (*C*=O), 154.6 (*C*), 152.0 (*C*), 143.9 (*C*), 126.1 (*C*H), 117.1 (*C*), 115.3 (*C*H), 114.0 (*C*H), 107.1 (*C*H), 18.7 (*C*H_3); ***m/z*** (ESI+, MeOH) 224 ($[M+Na]^+$, 100%), 202 ($[M+H]^+$, 7), 174 (37); **HRMS** (ESI+, MeOH) $[M+H]^+$ $C_{10}H_8O_2N_3$ requires 202.0611, found 202.0611; λ_{ex} = 350 nm.
^{1}H and ^{13}C NMR spectroscopic data in good agreement with the literature [43].

Azidomethyl-7-methoxy-chromen-2-one 11

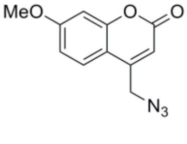

To a solution of 4-bromomethyl-7-methoxycoumarin (0.050 g, 0.18 mmol) in water (7 ml), was added NaN_3 (0.029 g, 0.45 mmol) and the solution was heated to 80 °C for 16 h. The aqueous layer was extracted with DCM (3 × 5 ml), the solvent was removed *in vacuo* and the crude residue was purified by column chromatography (DCM) to give azide **11** as a pale yellow solid (0.035 g, 82%). **R_f** (DCM:MeOH, 95:5) = 0.8; **mp** 142 – 144 °C; **IR** 2110 (N_3), 1716 (C=O), 1611 (C=C); **^{1}H NMR** δ (360 MHz, $CDCl_3$) 7.43 (1H, d, *J* = 8.8 Hz, Ar*H*), 6.89 – 6.83 (2H, m, Ar*H*), 6.35 (1H, t, *J* = 1.2 Hz, Ar*H*), 4.51 (2H, d, *J* = 1.2 Hz, CH_2), 3.87 (3H, s, OCH_3); **^{13}C NMR** δ (90.6 MHz, $CDCl_3$) 162.9 (*C*=O), 160.6 (*C*), 155.6 (*C*), 148.5 (*C*), 124.7 (*C*H), 112.6 (*C*H), 111.4 (*C*H), 110.7 (*C*), 101.2 (*C*H), 55.7 (*C*H_3), 50.7 (*C*H_2); ***m/z*** (FAB+, 3-NOBA), MeOH) 232 ($[M+H]^+$, 35%), 154 (100), 136 (92); **HRMS** (FAB+, 3-NOBA) $[M+H]^+$ $C_{11}H_{10}N_3O_3$ requires 232.0728, found 232.0726; λ_{ex} = 325 nm.
^{1}H and ^{13}C NMR spectroscopic data in good agreement with the literature [44].

7-Azido-4-methyl-1*H*-quinolin-2-one 12

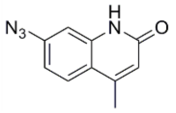

Carbostyril 124 (19.5 mg, 0.112 mmol) was dissolved in a solution of concentrated sulfuric acid (0.03 ml) and water (0.14 ml). The resultant solution was cooled to 0 ℃, and a solution of sodium nitrite (17.1 mg, 0.248 mmol) in water (0.1 ml) was added dropwise with stirring, whereupon a yellow slurry was formed. The solution was stirred at 0 ℃ for a further 15 min and a solution of sodium azide (22.8 mg, 0.350 mmol) in water (0.1 ml) was added with vigorous stirring and the solution became colourless. The mixture was stirred for 1 h at 0 ℃, then overnight at room temperature. The reaction mixture was basified with sat. Na_2CO_3 solution and extracted with DCM (3 × 1 ml). The combined organics were washed with water (2 × 1 ml), dried with $MgSO_4$, filtered and the solvent was removed *in vacuo*. The solid was recrystallised from hot ethanol to yield azide **12** as a pale yellow solid (22.4 mg, 65%). $\mathbf{R_f}$ (DCM:MeOH, 9:1) = 0.6; **mp** 190 ℃ (decomp); **IR** 2123 (N_3), 2098 (N_3), 1692 (C=O), 1660 (C=C), 1627; **^{1}H NMR** δ (500 MHz, DMSO-d$_6$) 11.56 (1H, s, N*H*), 7.72 (1H, d, *J* = 8.7 Hz, Ar*H*), 6.99 (1H, d, *J* = 2.2 Hz, Ar*H*), 6.94 (1H, dd, *J* = 8.6, 2.2 Hz, Ar*H*), 6.33 (1H, br s, Ar*H*), 2.39 (3H, s, C*H*$_3$); **^{13}C NMR** δ (126 MHz, DMSO-d$_6$) 161.7 (*C*=O), 147.7 (*C*), 141.3 (*C*), 139.9 (*C*), 126.0 (*C*H), 120.0 (*C*H), 117.1 (*C*), 113.1 (*C*H), 104.6 (*C*H), 18.4 (*C*H$_3$); ***m/z*** (ESI+, MeOH) 423 ($[2M+Na]^+$, 10%), 223 ($[M+Na]^+$, 35), 201 ($[M+H]^+$, 47), 173 (40), 145 (70), 130 (25); **HRMS** (ESI+, MeOH) $[M+H]^+$ $C_{10}H_9ON_4$ requires 201.0771, found 201.0777; λ_{ex} = 375 nm.

^{13}C NMR spectroscopic data in good agreement with the literature [48].

5-Dimethylamino-naphthalene-1-sulfonic acid (2-azido-ethyl)-amide 13

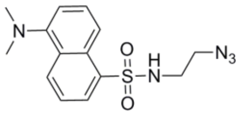

5-(Dimethylamino)naphthalene-1-sulfonyl chloride (2.50 g, 9.26 mmol) and 2-bromoethylamine hydrobromide (1.90 g, 9.26 mmol) were stirred at room temperature in DCM (46 ml) for 3 h in the presence of Et_3N (2.60 ml, 18.4 mmol). The solvent was removed *in vacuo* and the residue was dissolved in MeCN (46 ml). NaN_3 (1.48 g, 22.7 mmol) was added and the mixture heated to reflux overnight. After cooling to room temperature, the solvent was removed *in vacuo* and the crude residue was purified by column chromatography (Hexane:EtOAc, 1:1) to afford dansyl azide **13** as a green/yellow oil (2.06 g, 70%); $\mathbf{R_f}$ (DCM) = 0.3; **IR** 3300 (NH), 2105 (N_3); **^{1}H NMR** δ (250 MHz, $CDCl_3$) 8.54 (1H, d, *J* = 8.5 Hz, Ar*H*), 8.30 (1H, d, *J* = 8.7 Hz, Ar*H*), 8.24 (1H, dd, *J* = 7.3, 1.3 Hz, Ar*H*), 7.58 - 7.47 (2H, m, Ar*H*), 7.17 (1H, d, *J* = 7.5 Hz, Ar*H*), 5.54 (1H, t, *J* = 6.3 Hz, N*H*), 3.27 (2H, t, *J* = 5.8 Hz, C*H*$_2$), 3.05 (2H, q, *J* = 5.9 Hz, C*H*$_2$NH), 2.87 (6H, s, 2 × CH_3); **^{13}C NMR** δ (63 MHz, $CDCl_3$) 152.0 (*C*), 134.5 (*C*), 130.7 (*C*H), 129.9 (*C*), 129.5 (*C*H), 128.6 (*C*), 128.5 (*C*H), 123.1 (*C*H), 118.5 (*C*H), 115.3 (*C*H), 50.8 (*C*H$_2$), 45.3 (2 × *C*H$_3$), 42.3 (*C*H$_2$); ***m/z*** (ESI+, MeOH) 661 ($[2M+Na]^+$, 100%), 342 ($[M+Na]^+$, 80), 320 ($[M+H]^+$, 82).

^{1}H and ^{13}C NMR spectroscopic data in good agreement with the literature [49].

General procedure for the CuAAC reaction

General Procedure 2: Preparation of "Clicked" complexes

To Eu-DOTA complex **1a**, or Tb-DOTA complex **1b** (1 eq; dissolved at 20 mM concentration) in ^{t}BuOH:H_2O (2:1) was added TBTA (0.1 eq) and the mixture was allowed to stir for 15 min. Sodium ascorbate (0.2 eq; 0.1 M aq.) was added and the mixture was allowed to stir for 15 min followed by the addition of copper(II) sulfate (0.1 eq; 0.1 M aq.). After a further 15 min stirring the appropriate azide was added (1 eq) and the solution was allowed to stir under nitrogen at room temperature for 16 h. QuadraPure-IDA® metal scavenger resin was added and the mixture was gently shaken at room temperature overnight, during which the blue colour of the solution faded. The resin was removed by filtration and the solvent was then removed *in vacuo* to give the crude triazole sensor.

Spectroscopic data for sensors 14-21

Europium(III)-DOTA-6-triazole-methyl-pyridine-2-carboxylic acid methyl ester complex 14-Eu

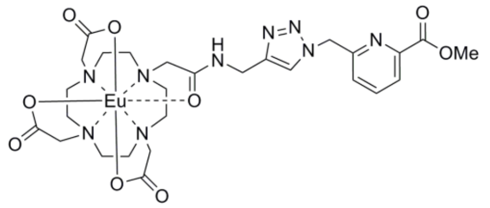

According to **general procedure 2**, Eu-DOTA complex **1a** (95.0 mg, 0.161 mmol), picolinate methyl ester **6** (31.6 mg, 0.161 mmol), TBTA (8.5 mg, 0.0161 mmol), NaAsc (320 µl, 0.032 mmol, 0.1 M aq.), $CuSO_4$ (160 µl, 0.016 mmol, 0.1 M aq.) and $^tBuOH:H_2O$ (8 ml, 2:1) afforded crude triazole **14-Eu** as a green/brown solid (110.0 mg, 89%). **IR** 3445 (OH/NH), 2106*, 1618 (C=O); **^{1}H NMR** δ (500 MHz, D_2O) 33.29 (s), 31.85 (s), 31.02 (s), 30.67 (s), 8.12 (s), 7.98 (s), 7.74 (s), 7.60 –7.25 (m), 6.89 (s), 5.76 (s), 5.58 (s), 4.99 (s), 4.73 (s), 4.20 – 3.75 (m), 3.62 (s), 1.31 (s), 0.10 (s), -0.27 (s), -2.44 (s), -2.77 (s), -3.22 (s), -4.29 (s), -4.63 (s), -5.60 (s), -5.76 (s), -7.14 (s), -7.56 (s), -7.91 (s), -11.10 (s), -11.47 – -11.66 (m), -12.36 (s), -14.57 (s), -15.07 (s), -15.78 (s), -16.70 (s), -17.06 (s); ***m/z*** (ESI+, MeOH) 822 ([^{153}EuM+K]$^+$, 40%), 820 ([^{151}EuM+K]$^+$, 36), 806 ([^{153}EuM+Na]$^+$, 30), 804 ([^{151}EuM+Na])$^+$, 28), 784 ([^{153}EuM+H]$^+$, 32), 782 ([^{151}EuM+H]$^+$, 30), 414 (45), 344 (38), 284 (65); **HRMS** (ESI+, H_2O) [^{153}EuM+H]$^+$ $C_{27}H_{36}{}^{151}EuN_9O_9$ requires 784.1926, found 784.1928, [^{151}EuM+H]$^+$ $C_{27}H_{36}{}^{153}EuN_9O_9$ requires 782.1912, found 782.1920, λ_{ex} = 325 nm.

*From starting material

Terbium(III)-DOTA-6-triazole-methyl-pyridine-2-carboxylic acid methyl ester complex 14-Tb

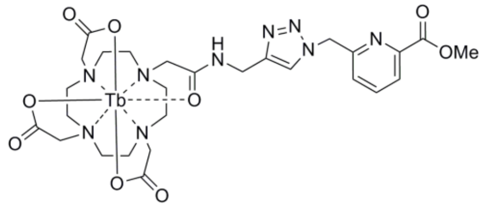

According to **general procedure 2**, Tb-DOTA complex **1b** (50.0 mg, 0.0837 mmol), picolinate methyl ester **6** (11.2 mg, 0.0563 mmol), TBTA (4.4 mg, 8.37 µmol), NaAsc (170 µl, 16.7 µmol, 0.1 M aq.) $CuSO_4$ (84 µl, 8.37 µmol, 0.1 M aq.) and $^tBuOH:H_2O$ (6 ml, 2:1) afforded crude triazole **14-Tb** as a pale brown solid (60.0 mg, 91%). **IR** 3426 (OH/NH), 2107*, 1729 (C=O), 1626 (C=O), 1592 (C=C); **^{1}H NMR** δ (600 MHz, D_2O) 258.1 (s), 241.0 – 237.1 (m), 209.8 (s), 201.8 (s), 111.8 – 109.3 (m), 59.0 (s), 46.8 – 44.5 (m), 17.9 (s), 17.2 (s), 16.7 (s), 15.4 (s), 12.9 (s), 10.5 (s), 8.3 (s), 8.0 (s), 7.8 (s), 6.0 – 5.8 (m), 4.9 – 3.0 (m), 2.4 (s), 1.5 – 1.4 (m), 1.0 – -2.0 (m), -67.2 (s), -73.5 (s), -76.9 (s), -105.5 (s), -113.6 – -114.6 (m), -126.1 (s), -192.2 – 200.2 (m), -210.3 (s) -365.6 (s), -373.4 – -376.8 (m); ***m/z*** (ESI+) 828 ([M+K]$^+$, 100%), 812 ([M+Na]$^+$, 77), 790 ([M+H]$^+$, 5); **HRMS** (ESI–, MeOH) [M-H]$^-$ $C_{27}H_{35}N_9O_9Tb$ requires 788.1817, found 788.1822; λ_{ex} = 325 nm.

*From starting material

Europium(III)-DOTA-6-triazole-methyl-pyridine-2-carboxylic acid complex 15-Eu

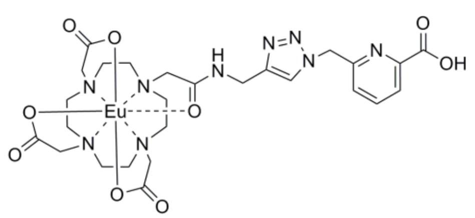

According to **general procedure 2**, Eu-DOTA complex **1a** (40.0 mg, 0.0676 mmol), picolinate carboxylic acid **7** (13.7 mg, 0.0676 mmol), TBTA (3.5 mg, 6.76 µmol), NaAsc (135 µl, 13.5 µmol, 0.1 M aq.), $CuSO_4$ (68 µl, 6.76 µmol, 0.1 M aq.) and $^tBuOH:H_2O$ (3 ml, 2:1) afforded crude triazole **15-Eu** as a green solid (42.6 mg, 82%). **IR** (KBr disk) 3423 (OH/NH), 3115 (OH/NH), 2123*, 1623 (C=O); **^{1}H NMR** δ (360 MHz, D_2O) 32.56 (s), 31.27 (s), 30.44 (s), 30.08 (s), 8.23 – 7.40 (m), 4.94 – 0.85 (m), -0.05 (s), -0.39 (s), -2.38 (s), -3.36 (s), -4.16 (s), -5.76 (s), -7.14 (s), -7.72 (s), -8.07 (s), -10.61 (s), -11.23 (s), -11.57 (s), -12.21 (s), -14.01 (s), -13.96 – -15.34 (m), -15.44 (s), -16.44 (s); ***m/z*** (ESI+, H_2O) 770 ([^{153}EuM+H]$^+$), 768 ([^{151}EuM+H]$^+$); **HRMS** (FAB, 3-NOBA) $C_{26}H_{35}{}^{153}EuN_9O_9$ requires 770.1770, found 770.1779; $C_{26}H_{35}{}^{151}EuN_9O_9$ requires 768.1756, found 768.1764; λ_{ex} = 325 nm.

*From starting material

Terbium(III)-DOTA-6-triazole-methyl-pyridine-2-carboxylic acid complex 15-Tb

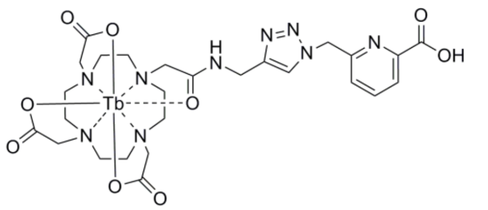

According to **general procedure 2**, Tb-DOTA complex **1b** (50.0 mg, 0.0837 mmol), carboxylic acid **7** (16.1 mg, 0.0837 mmol), TBTA (4.4 mg, 8.37 μmol), NaAsc (170 μl, 16.7 μmol, 0.1 M aq.) $CuSO_4$ (84 μl, 8.37 μmol, 0.1 M aq.) and $^tBuOH:H_2O$ (6 ml, 2:1) afforded crude triazole **15-Tb** as a pale brown solid (57.6 mg, 89%). **IR** (KBr disc) 3446 (OH/NH), 2118*, 1626 (C=O); **^{1}H NMR** δ (600 MHz, D_2O) 256.2 (s), 242.2 – 236.2 (m), 215.2 (s), 198.4 (s), 115.6 (s), 108.5 (s), 60.7 (s), 45.2 – 43.3 (m), 19.8 (s), 18.4 (s), 17.5 (s), 13.3 (s), 11.5 (s), 10.3 (s), 8.6 – 8.2 (m), 7.7 – 7.3 (m), 7.3 – 7.2 (m), 5.2 – 4.5 (m), 4.3 – 3.5 (m), 2.6 (s), 1.7 – 1.4 (m), -63.4 (s), -75.4 (s), -103.3 (s), -113.8 (s), -116.4 (s), -126.9 (s), -190.2 – 200.6 (m), -362.8 (s), -377.0 (s); ***m/z*** (ESI–, MeOH) 774 ($[M-H]^-$, 81%), 632 (47), 596 (76), 440 (100); **HRMS** (ESI–, MeOH) $[M-H]^-$ $C_{26}H_{33}N_9O_9Tb$ requires 774.1660, found 774.1614; λ_{ex} = 325 nm.

*From starting material

Europium(III)-DOTA-triazole-picolinate-carboxylic acid methyl ester complex 16-Eu

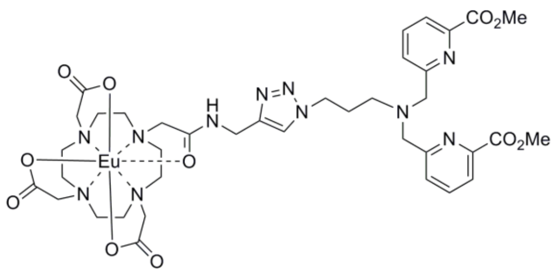

According to **general procedure 2**, Eu-DOTA complex **1a** (88.0 mg, 0.149 mmol), picolinate ester **8** (59.4 mg, 0.149 mmol), TBTA (7.9 mg, 0.0149 mmol), NaAsc (298 μl, 0.0298 mmol, 0.1 M aq.), $CuSO_4$ (149 μl, 0.0149 mmol, 0.1 M aq.) and $^tBuOH:H_2O$ (15 ml, 2:1) afforded crude triazole **16-Eu** as a green solid (115.0 mg, 78%). **IR** (KBr disk) 3564 (OH/NH), 2102*, 1739 (C=O), 1616 (C=O); **^{1}H NMR** δ (600 MHz, CD_3OD) 38.54 – 37.77 (m), 35.96 – 35.03 (m), 34.84 – 33.97 (m), 33.89 – 33.00 (m), 17.01 – 15.90 (m), 14.68 – 13.26 (m), 8.48 – 8.29 (m), 7.88 (d, J = 52.9 Hz), 7.61 (s), 7.45 (s), 7.27 (s), 5.50 (s), 3.92 (d, J = 32.4 Hz), 3.71 (s), 3.27 (s), 2.77 – 2.19 (m), 1.78 (s), 1.51 (s), 1.23 (s), 0.84 (s), 0.60 – 0.29 (m), -0.28 – -0.53 (m), -0.97 – -1.22 (m), -1.72 – -2.12 (m), -2.58 – -3.05 (m), -3.45 (s), -4.42 – -4.96 (m), -6.69 (s), -10.55 – -11.27 (m), -12.48 – -12.80 (m), -13.70 – -14.57 (m), -16.93 (s), -17.99 (s). ***m/z*** (ESI+, H_2O) 1028 ($[^{153}EuM+K]^+$, 20%), 1026 ($[^{151}EuM+K]^+$, 22), 1012 ($[^{153}EuM+Na]^+$, 25), 1010 ($[^{151}EuM+Na]^+$, 30), 990 ($[^{153}EuM+H]^+$, 85), 988 ($[^{151}EuM+H]^+$, 100) 964 (50); **HRMS** (FAB, 3-NOBA) $[^{153}EuM+Na]^+$ $C_{38}H_{50}{}^{153}EuN_{11}O_{11}Na$ requires 1012.2801 found 1012.2763, $[^{151}EuM+Na]^+$ $C_{38}H_{50}{}^{151}EuN_{11}O_{11}Na$ requires 1010.2788, found 1010.2755; λ_{ex} = 300 nm.

*From starting material

Terbium(III)-DOTA-triazole-picolinate-carboxylic acid methyl ester complex 16-Tb

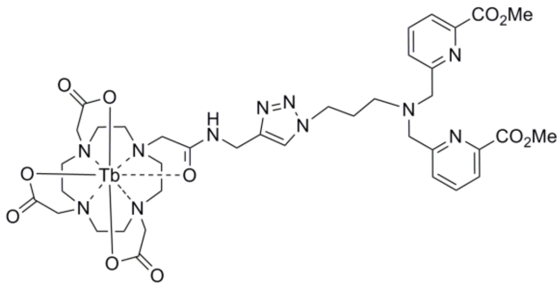

According to **general procedure 2**, Tb-DOTA complex **1b** (50.0 mg, 0.0837 mmol), methyl ester **8** (33.4 mg, 0.0837 mmol), TBTA (4.4 mg, 8.37 μmol), NaAsc (170 μl, 16.7 μmol, 0.1 M aq.), $CuSO_4$ (84 μl, 8.37 μmol, 0.1 M aq.) and $^tBuOH:H_2O$ (6 ml, 2:1) afforded crude triazole **16-Tb** as a colourless solid (75.7 mg, 91%). **IR** 3506 (OH/NH), 2103*, 1728 (C=O), 1592 (C=C); **^{1}H NMR** δ (600 MHz, D_2O) 256.4 (s), 243.3 (s), 236.6 (s), 213.9 (s), 199.5 (s), 115.7 (s), 108.8 (s), 60.4 (s), 44.8 – 42.9 (m), 19.7 (s), 15.2 (s), 14.6 (m), 11.4 (s), 9.6 (s), 9.0 , 8.7 – 8.5 (m), 8.3 – 8.2 (m), 7.8 (s), 7.6 – 6.9 (m), 6.1 (s), 5.8 (s), 5.0 – 4.8 (m), 4.5 – 3.2 (m), 2.5 (m), 2.2 – 1.4 (m), -63.3 (s), -75.4 (s), -103.1 (s), -111.2 (s), -116.8 (s), -127.3 (s), -190.5 (s), -363.3 (s), -376.8 (s); ***m/z*** (ESI–, MeOH) 994 ($[M-H]^-$, 11%), 337 (13), 149 (100); **HRMS** (ESI–, MeOH) $[M-H]^-$ $C_{38}H_{49}N_{11}O_{11}Tb$ requires 994.2872, found 994.2824; λ_{ex} = 300 nm.

*From starting material

Europium(III)-DOTA-triazole-picolinate-carboxylic acid complex 17-Eu

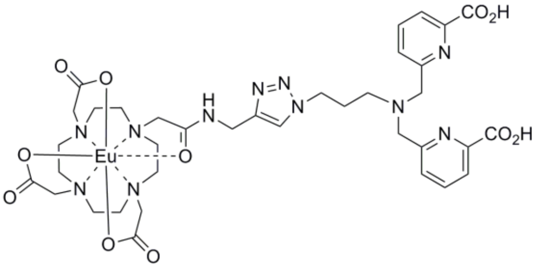

According to **general procedure 2**, Eu-DOTA complex **1a** (95.0 mg, 0.160 mmol), carboxylic acid **9** (59.4 mg, 0.160 mmol), TBTA (8.5 mg, 0.0160 mmol), NaAsc (320 µl, 0.0320 mmol, 0.1 M aq.), $CuSO_4$ (160 µl, 0.0160 mmol, 0.1 M aq.) and $^tBuOH:H_2O$ (8 ml, 2:1) afforded crude triazole **17-Eu** as a brown solid (130.5 mg, 85%). **IR** (KBr disk) 3455 (OH/NH), 2104*, 1751 (C=O), 1623 (C=O); **^{1}H NMR** δ (600 MHz, CD_3OD) δ 33.90 – 33.08 (m), 32.55 – 31.44 (m), 31.33 – 30.98 (m), 30.90 – 30.12 (m), 11.31 – 9.70 (m), 7.99 (d, J = 51.9 Hz), 7.63 (s), 7.26 (s), 5.54 (s), 3.46 (s), 3.23 (s), 2.11 (s), 1.43 – 1.11 (m), 0.81 (s), 0.29 – -0.22 (m), -0.29 – -0.87 (m), -2.35 – -4.09 (m), -4.92 – -5.54 (m), -6.91 – -7.61 (m), -10.19 – -11.16 (m), -11.39 – -13.16 (m), -14.30 – -14.98 (m), -14.95 – -15.72 (m), -15.74 – -16.36 (m), -16.95 – -17.57 (m); ***m/z*** (ESI+, H_2O) 984 ([^{153}EuM+Na]$^+$, 12%), 982 ([^{151}EuM+Na]$^+$, 10), 531 (100); **HRMS** (FAB, 3-NOBA) [^{153}EuM+Na]$^+$ $C_{36}H_{46}{}^{153}EuN_{11}O_{11}Na$ requires 984.2488, found 984.2480; λ_{ex} = 300 nm.

Terbium(III)-DOTA-triazole-picolinate-carboxylic acid complex 17-Tb

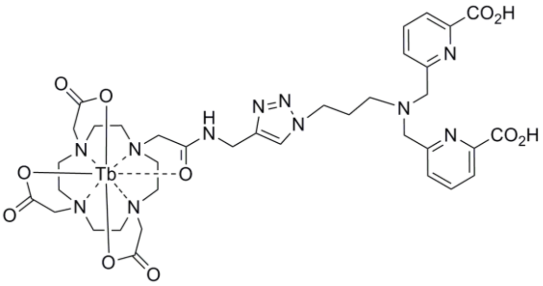

According to **general procedure 2**, Tb-DOTA complex **1b** (50.0 mg, 0.0837 mmol), carboxylic acid **9** (30.9 mg, 0.0837 mmol), TBTA (4.4 mg, 8.37 µmol), NaAsc (170 µl, 16.7 µmol, 0.1 M aq.) $CuSO_4$ (84 µl, 8.37 µmol, 0.1 M aq.) and $^tBuOH:H_2O$ (6 ml, 2:1) afforded crude triazole **17-Tb** as a pale brown solid (69.5 mg, 86%). **IR** (KBr disc) 3443 (OH/NH), 2104*, 1748 (C=O), 1634 (C=O), 1598 (C=C); **^{1}H NMR** δ (600 MHz, D_2O) 254.9 (s), 239.8 (s), 235.1 (s), 210.0 (s), 196.4 (s), 113.9 (s), 107.4 (s), 60.8 (s), 49.1 (s), 44.8 – 43.3 (m), 19.6 (s), 12.5 (s), 8.1 – 8.0 (m), 7.7 (s), 5.5 (s), 4.8 – 4.7 (m), 4.3 (s), 4.2 (s), 4.1 (s) 3.7 – 3.6 (m), 3.4 (s), 3.1 (s), 2.9 – 2.7 (m), 2.3 (s), 2.1 – 2.0 (m), 1.2 (s), -63.5 (s), -75.0 (s), -102.9 (s), -113.1 (s), -115.6 (s), -125.3 (s), -360.9 (s), -374.4 (s); ***m/z***; (ESI–, MeOH) 966 ([M-H]$^-$, 5%) 631 (46), 596 (28), 336 (16), 148 (100); **HRMS** (ESI–, MeOH) [M-H]$^-$ $C_{36}H_{45}N_{11}O_{11}Tb$ requires 966.2559, found 966.2500; λ_{ex} = 300 nm.

*From starting material

Europium(III)-DOTA-7-triazole-4-methyl-chromen-2-one complex 18-Eu

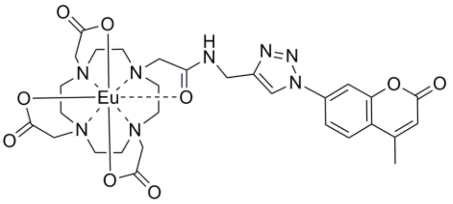

According to **general procedure 2**, Eu-DOTA complex **1a** (87.9 mg, 0.149 mmol), coumarin azide **10** (29.9 mg, 0.149 mmol), TBTA (7.9 mg, 0.0149 mmol), NaAsc (298 µl, 0.0298 mmol, 0.1 M aq.), $CuSO_4$ (149 µl, 0.0149 mmol, 0.1 M aq.) and $^tBuOH:H_2O$ (15 ml, 2:1) afforded crude triazole **18-Eu** as a green solid (88.6 mg, 75%). **IR** (KBr disk) 3483 (OH/NH), 2121*, 1815 (C=O), 1726 (C=O), 1614 (C=C); **^{1}H NMR** δ (600 MHz, D_2O) 33.00 (s), 31.48 (s), 30.79 (s), 8.78 (s), 7.87 (s), 7.62 (d, J = 66.9 Hz), 7.20 (d, J = 67.2 Hz), 6.97 (d, J = 36.5 Hz), 6.38 (s), 6.22 (s), 5.26 (s), 4.75 (d, J = 52.2 Hz), 4.29 (s), 4.20 (s), 4.02 (s), 2.47 (s), 2.38 (d, J = 12.1 Hz), 2.27 (s), 0.11 (s), -0.18 (s), -2.47 (s), -3.20 (s), -4.23 (s), -5.70 (s), -7.09 (s), -7.36 (s), -7.81 (s), -11.25 (s), -11.02 (s) -11.53 (s), -12.10 (s), -14.06 (s), -14.33 (s), -14.84 (s), -15.64 (s), -16.70 (s); ***m/z*** (ESI+, H_2O), 831 ([^{153}EuM+K]$^+$ 28%), 829 ([^{151}EuM+K]$^+$, 26), 793 ([^{153}EuM+H]$^+$, 27), 791 ([^{151}EuM+H]$^+$, 30); **HRMS** (FAB, 3-NOBA) $C_{29}H_{36}{}^{153}EuN_8O_9$ [^{153}EuM+H]$^+$ requires 793.1818, found 793.1821; [^{151}EuM+H]$^+$ $C_{29}H^{151}Eu_{36}N_8O_9$ requires 791.1804, found 791.1815; λ_{ex} = 345 nm.

* From starting material

Terbium(III)-DOTA-7-triazole-4-methyl-chromen-2-one complex 18-Tb

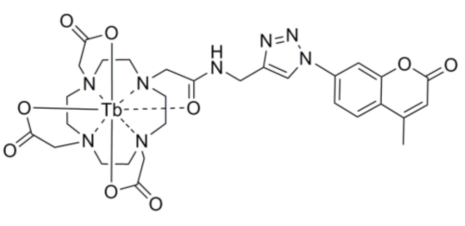

According to **general procedure 2**, Tb-DOTA complex **1b** (50.0 mg, 0.0837 mmol), coumarin azide **10** (16.8 mg, 0.0837 mmol), TBTA (4.4 mg, 8.37 μmol), NaAsc (170 μl, 16.7 μmol, 0.1 M aq.), $CuSO_4$ (84 μl, 8.37 μmol, 0.1 M aq.) and $^tBuOH:H_2O$ (6 ml, 2:1) afforded crude triazole **18-Tb** as a colourless residue (58.0 mg, 87%). **IR** (KBr disc) 3441 (OH/NH), 2120*, 1717 (C=O), 1616 (C=O); **^{1}H NMR** δ (600 MHz, D_2O) 256.2 (s), 242.5 (s), 237.9 (s), 212.3 (s), 200.8 (s), 115.0 – 110.1 (m), 47.7 – 42.7 (m), 19.8 (s), 14.9 (s), 14.2 (s), 12.8 (s), 11.6 (s), 9.2 (s), 8.1 – 7.6 (m), 5.8 (s), 3.8 (s), 3.5 (s), 3.2 (s), 3.0 – 2.1 (m), 1.8 – 1.4 (m), -63.1 – -65.4 (m), -72.7 – -74.8 (m). -63.5 – -65.6 (m), -73.8 – -77.1 (m), -102.7 –-105.7 (m), -113.6 – -118.1 (m), -127.8 – 129.5 (m), -195.2 (s) -363.0 – -365.9 (m), -376.5 – -379.8 (m); ***m/z*** (ESI–, MeOH) 797 ([M-H]$^-$, 54%), 265 (100); **HRMS** (ESI–, MeOH) [M-H]$^-$ $C_{29}H_{34}N_8O_9Tb$ requires 797.1708, found 797.1662; λ_{ex} = 345 nm.

*From starting material

Europium(III)-DOTA-4-triazolemethyl-7-methoxy-chromen-2-one complex 19-Eu

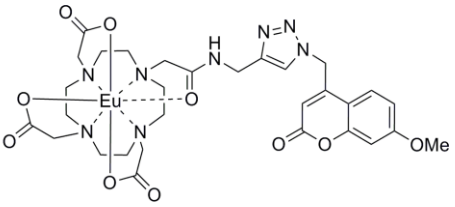

According to **general procedure 2**, Eu-DOTA complex **1a** (51.0 mg, 0.0862 mmol), coumarin azide **11** (19.8 mg, 0.0862 mmol), TBTA (4.6 mg, 8.62 μmol), NaAsc (172 μl, 0.00172 mmol, 0.1 M aq.), $CuSO_4$ (86 μl, 8.62 μmol, 0.1 M aq.) and $^tBuOH:H_2O$ (6 ml, 4:2) afforded crude triazole **19-Eu** as a brown solid (56.0 mg, 79%$^\wedge$).$^\$$ **IR** (KBr disk) 3427 (OH/NH), 2116*, 1685 (C=O), 1612 (C=O); **^{1}H NMR** δ (600 MHz, CD_3OD) 37.99 (s), 35.65 – 35.38 (m), 34.37 (s), 33.60 (s), 16.50 (s), 14.04 – 13.14 (m), 8.40 (s), 7.27 (s), 6.80 (s), 5.95 – 4.91 (m), 3.98 – 3.51 (m), 3.43 – 3.07 (m), 2.29 (s), 1.23 (s), 0.84 (s), 0.56 (s), -1.06 (s), -1.85 (s), -2.74 (s), -3.62 (s), -4.74 (s), -6.63 (s), -11.06 (s), -12.56 (s), -14.03 (s), -16.85 (s), -17.32 – -18.66 (m); ***m/z*** (ESI+, H_2O) 861 ([^{153}EuM+K]$^+$, 35%), 859 ([^{151}EuM+K]$^+$, 34), 845 ([^{153}EuM+Na]$^+$, 55%), 843 ([^{151}EuM+Na]$^+$, 58), 823 ([^{153}EuM+H]$^+$, 100), 821 ([^{151}EuM+H]$^+$, 87); **HRMS** (FAB, 3-NOBA) $C_{30}H_{38}{}^{151}EuN_8O_{10}$ requires 821.1910, found 821.1889; λ_{ex} = 325 nm.

$^\wedge$ % recovered

$^\$$ 84% conversion to triazole – calculation from IR analysis

*From starting material

Terbium(III)-DOTA-4-triazolemethyl-7-methoxy-chromen-2-one complex 19-Tb

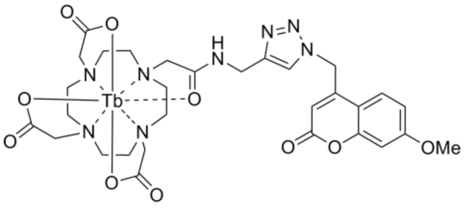

According to **general procedure 2**, Tb-DOTA complex **1b** (50.0 mg, 0.0837 mmol), coumarin azide **11** (19.3 mg, 0.0837 mmol), TBTA (4.4 mg, 8.37 μmol), NaAsc (170 μl, 16.7 μmol, 0.1 M aq.), $CuSO_4$ (84 μl, 8.37 μmol, 0.1 M aq.) and $^tBuOH:H_2O$ (6 ml, 2:1) afforded crude triazole **19-Tb** as a pale blue solid (49.2 mg, 71%$^\wedge$).$^\$$ **IR** (KBr disc) 3422 (OH/NH), 2115*, 1716 (C=O), 1615 (C=O); **^{1}H NMR** δ (600 MHz, D_2O) 254.3 (s), 239.2 – 232.8 (m), 207.6 (s), 200.8 (s), 182.5 – 180.0 (m), 165.4 (s) 112.2 (s), 108.9 (s), 46.0 – 43.8 (m), 19.2 – 18.7 (m), 17.9 (s), 15.6 (s), 11.9 (s), 11.1 (s), 9.8 (s), 8.5 – 8.3 (m), 7.8 – 6.4 (m), 5.7 (s), 5.5 (s), 4.6 – 2.5 (s), 2.4 (s), 2.3 – 1.5 (m), 1.5 (s), -1.0 (s), -2.0 (s), -14.2 (s), -15.5 – 44.6 (m), -66.5 (s), -73.2 (s), -76.3 (s), -86.2 (s), -104.9 (s), -113.0 – 115.1 (m), -125.3 (s), -145.5 (s), -154.4 (s), -190.5 (s), -325.0 (s), -340.5 (s) -363.6 (s), -372.0 (s), -376.2 (s), -385.2 (s), -395.6 (s); ***m/z*** (ESI–, MeOH) 827 ([M-H]$^-$, 67%), 596 (3); **HRMS** (ESI+, MeOH) [M+H]$^+$ $C_{30}H_{38}N_8O_{10}Tb$ requires 829.1959, found 829.1967; λ_{ex} = 325 nm.

The crude product was then purified by reverse phase HPLC using a gradient elution programme of 5 → 65% (B) over 70 min was used where (A) = H_2O + 0.1% TFA; (B) = MeCN + 0.1% TFA. R_t = 39.7 min. Triazole **19-Tb** was obtained as a pale blue solid (28.4 mg, 41%); **IR** (KBr disc) 3431 (OH/NH), 1718 (C=O), 1616 (C=O); ***m/z*** (ESI+, MeOH) 851 ([M+K]$^+$, 17%) 829 ([M+H]$^+$, 45), 715 (12), 690 (23), 304 (20).

$^\wedge$ % recovered

$^\$$ 85% conversion to triazole – calculation from IR analysis

* From starting material

Europium(III)-DOTA-7-triazole-4-methyl-1*H*-quinolin-2-one complex 20-Eu

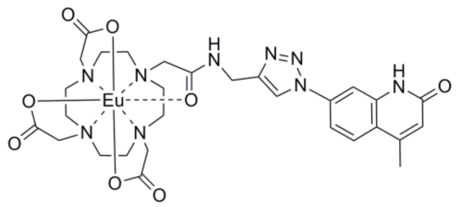

According to **general procedure 2**, Eu-DOTA complex **1a** (33.3 mg, 0.0563 mmol), carbostyril azide **12** (11.3 mg, 0.0563 mmol), TBTA (3.0 mg, 5.63 μmol), NaAsc (113 μl, 0.0113 mmol, 0.1 M aq.) $CuSO_4$ (56 μl, 5.63 μmol, 0.1 M aq.) and $^tBuOH:H_2O$ (3 ml, 2:1) afforded crude triazole **20-Eu** as a green solid (32.1 mg, 72%^).$ **IR** (KBr disk) 3411 (OH/NH), 2121*, 1674 (C=O), 1624 (C=O); ***m/z*** (ESI+, MeOH) 814 [^{153}EuM+Na]$^+$, 37%), 812 ([^{151}EuM+Na]$^+$, 39), 792 [^{153}EuM+H]$^+$, 55), 790 ([^{151}EuM+H]$^+$, 62); **^{1}H NMR** δ (600 MHz, D_2O) 32.95 (s), 31.40 (s), 30.61 (s), 12.02 – 10.51 (m), 9.11 – 8.91 (m), 7.85 – 6.24 (m), 4.61 (s), 4.44 (s), 4.29 (s), 4.19 (s), 3.76 – 3.65 (m), 3.61 (s), 3.37 (d, *J* = 12.5 Hz), 3.24 (s), 3.00 (d, *J* = 13.4 Hz), 2.79 (d, *J* = 13.2 Hz), 2.31 (s), 1.87 (s), 1.72 (s), 1.56 (s), 1.43 (s), 1.19 (s), 0.21 – -0.08 (m), -0.09 – -0.38 (m), -2.23 – -2.66 (m), -3.07 – -3.46 (m), -4.04 – -4.43 (m), -5.36 – -6.17 (m), -6.98 – -7.28 (m), -7.28 – -7.53 (m), -7.81 – -8.14 (m), -10.71 – -11.72 (m), -11.90 – -12.46 (m), -14.74 – 14.98 (m), -15.55 (s), -16.69 (s); **HRMS** (FAB, 3-NOBA) [^{153}EuM+H]$^+$ $C_{29}H_{37}{}^{153}EuN_9O_8$ requires 792.1978, found 792.1979; [^{151}EuM+H]$^+$ $C_{29}H_{37}{}^{151}Eu\ N_9O_8$ requires 790.1964, found 790.1977; λ_{ex} = 345 nm.

^ % recovered

$ 83% conversion to triazole – calculation from IR analysis

* From starting material

Terbium(III)-DOTA-7-triazole-4-methyl-1*H*-quinolin-2-one complex 20-Tb

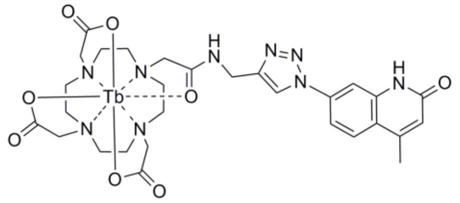

According to **general procedure 2**, Tb-DOTA complex **1b** (50.0 mg, 0.0837 mmol), carbostyril azide **12** (16.7 mg, 0.0837 mmol), TBTA (4.4 mg, 8.37 μmol), NaAsc (170 μl, 16.7 μmol, 0.1 M aq.) $CuSO_4$ (84 μl, 8.37 μmol, 0.1 M aq.) and $^tBuOH:H_2O$ (6 ml, 2:1) afforded crude triazole **20-Tb** as a colourless residue (49.9 mg, 75%^).$ **IR** (KBr disc) 3434 (OH/NH), 2120*, 1656 (C=O), 1626 (C=O); **^{1}H NMR** δ (600 MHz, D_2O) 255.7 (s), 241.8 (s), 236.4 (s), 211.7 – 197.9 (m), 115.2 – 108.8 (m), 59.2 (s), 44.7 – 43.1 (m), 19.8 (s), 14.9 (s), 14.2 (s), 12.8 (s), 11.3 (s), 9.2 (s), 8.5 – 7.6 (m), 5.8 (s), 3.8 (s), 3.5 (s), 3.2 (s), 2.9 – 2.5 (m), 2.0 (s), 1.4 – 1.0 (m), -62.6 – -65.6 (m), -72.9 – -74.8 (m), -102.9 – -105.5 (m), -113.4 – -116.4 (m), -127.6 (s), -362.6 (s), -375.6 (s); ***m/z*** (ESI–, MeOH) 796 ([M-H]$^-$, 100%), 596 (40), 265 (52); **HRMS** (ESI–, MeOH) [M-H]$^-$ $C_{29}H_{35}N_9O_8Tb$ requires 796.1868, found 796.1821; λ_{ex} = 345 nm.

^ % recovered

$ 68% conversion to triazole – calculation from IR analysis

* From starting material

Terbium(III)-DOTA-6-triazole-5-Dimethylamino-naphthalene-1-sulfonic acid (2-azido-ethyl)-amide 21-Tb

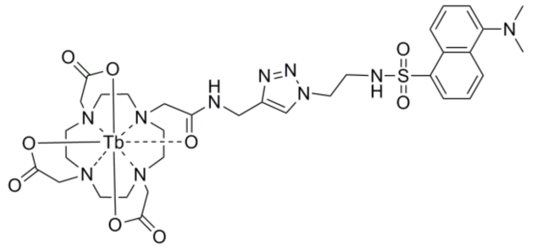

According to **general procedure 2**, Tb-DOTA complex **1b** (60.0 mg, 0.100 mmol), dansyl azide **13** (32.0 mg, 0.100 mmol), TBTA (5.3 mg, 10.0 μmol), NaAsc (200 μl, 20.0 μmol, 0.1 M, aq), $CuSO_4$ (100 μl, 0.0100 mmol, 0.1 M aq.) and $^tBuOH:H_2O$ (6 ml, 2:1) afforded crude triazole **21-Tb** as a pale yellow/green solid (90.5 mg, 98%). **IR** (KBr disc) 3444 (OH/NH), 2110*, 1683 (C=O), 1624 (C=O), 1259 (S=O), 1165 (S=O); **^{1}H NMR** δ (600 MHz, D_2O) 250.9 – 234.9 (m), 206.5 (s), 194.9 (s), 114.3 (s), 109.7 (s), 64.2 (s), 52.5 (s), 44.5 – 41.4 (m), 16.5 (s), 15.1 (s), 11.9 (s), 9.7 (s), 8.8 – 7.43 (m), 8.4 – 7.7 (m), 6.8 (s), 6.3(s), 4.4 –0.84 (m), -0.0 (s), -4.4 (s), -65.6 (s), -75.2 – 79.9 (m), -101.7 (s), -111.0 (s), -126.0 (s), -190.0 (s), 199.8 (s), -364.4 (s), -376.3 (s); ***m/z*** (ESI–, MeOH) 915 ([M-H]$^-$, 100%), 596 (2); **HRMS** (ESI–, MeOH) [M-H]$^-$ found 915.2267, $C_{33}H_{44}N_{10}O_9Tb$ requires 915.2272; λ_{ex} = 350 nm.

*From starting material

Luminescence spectra for sensors 14-21

<table>
<tr><th>Triazole Complex</th><th>Luminescence response</th></tr>
<tr><td>14-Eu
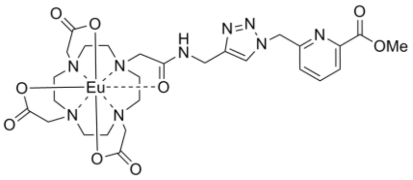
λex = 325 nm</td><td>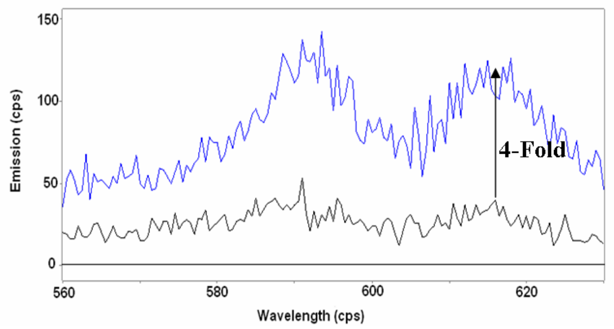
</td></tr>
<tr><td>14-Tb
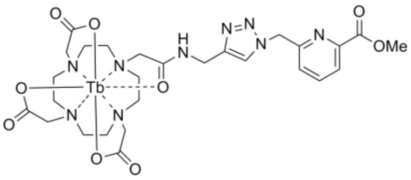
λex = 325 nm</td><td>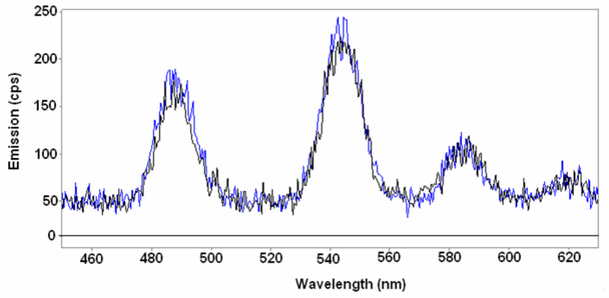
</td></tr>
<tr><td>15-Eu
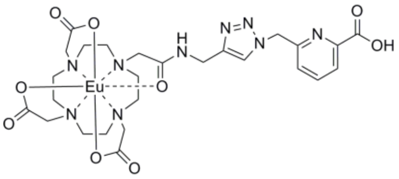
λex = 325 nm</td><td>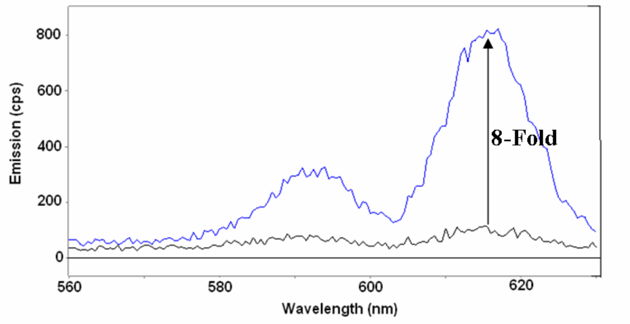
</td></tr>
<tr><td>15-Tb
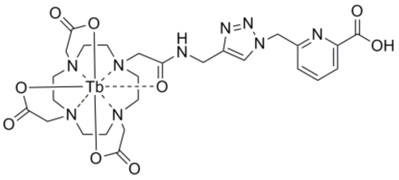
λex = 325 nm</td><td>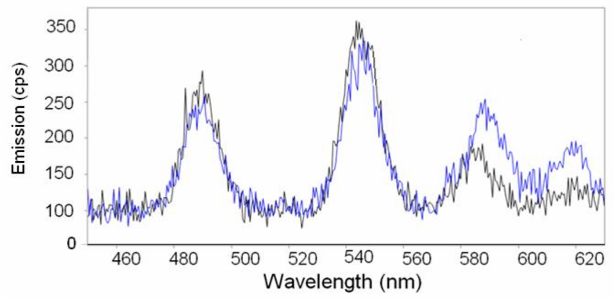
</td></tr>
<tr><td colspan="2">Table S1. Lanthanide luminescence of lanthanide-picolinate complexes 14-Eu/Tb and 15-Eu/Tb. The black line is the control experiment of lanthanide-DOTA-alkyne and fluorophore azide (both 100 μM) in the absence of copper. The blue line is 100 μM solution of crude lanthanide-triazole-fluorophore. (time delay = 0.076 ms, slits = 10 nm, sample window = 5 ms, number of flashes = 20)</td></tr>
</table>

<table>
<tr><th>Triazole Complex</th><th>Luminescence response</th></tr>
<tr><td>16-Eu
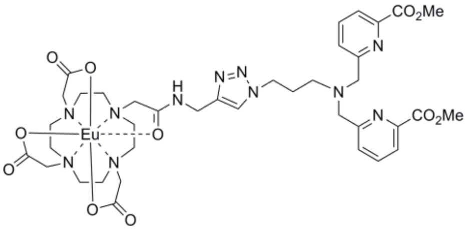

λ_{ex} = 300 nm</td><td>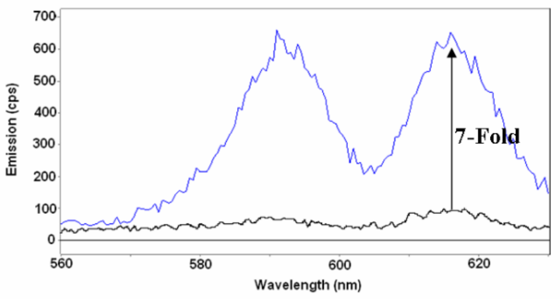
</td></tr>
<tr><td>16-Tb
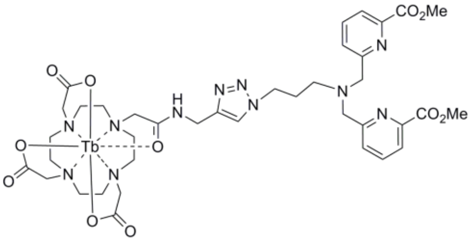

λ_{ex} = 300 nm</td><td>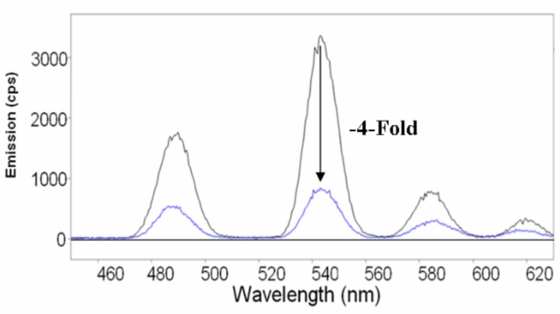
</td></tr>
<tr><td>17-Eu
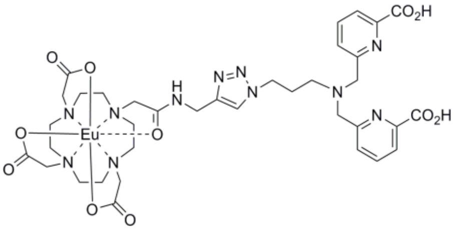

λ_{ex} = 300 nm</td><td>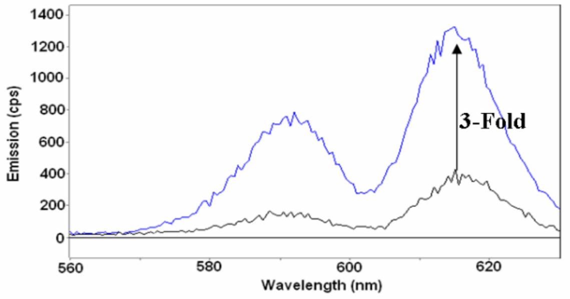
</td></tr>
<tr><td>17-Tb
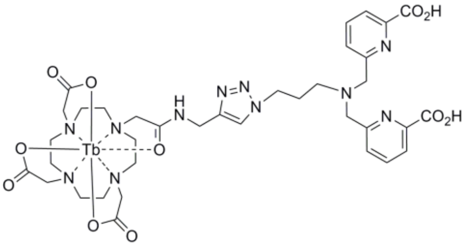

λ_{ex} = 300 nm</td><td>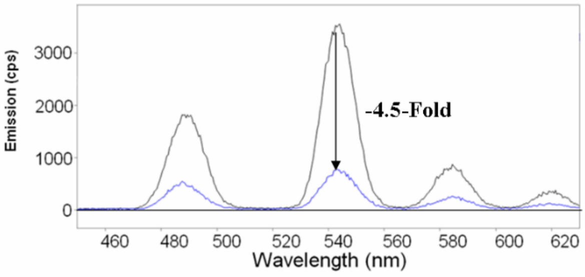
</td></tr>
</table>

Table S2. Lanthanide luminescence picolinate complexes **16-Eu/Tb** and **17-Eu/Tb**; The black line is the control experiment of lanthanide-DOTA-alkyne and fluorophore azide (both 100 μM) in the absence of copper, the blue line is 100 μM solution of crude lanthanide-triazole-fluorophore after CuAAC reaction. (time delay = 0.076 ms, slits = 10 nm, sample window = 5 ms, number of flashes = 20)

Triazole Complex	Luminescence response
**18-Eu**<br>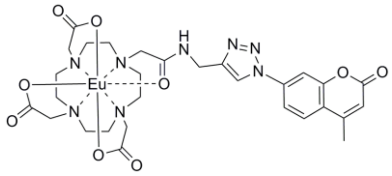<br><br>λ_{ex} = 345 nm	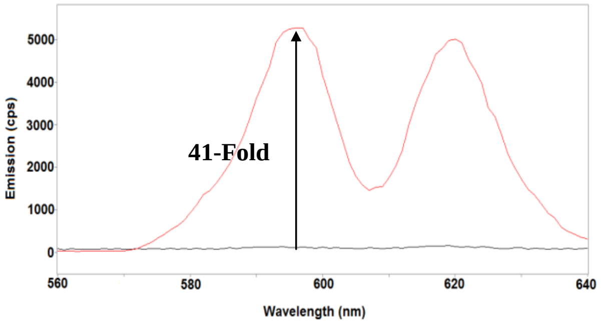
**18-Tb**<br>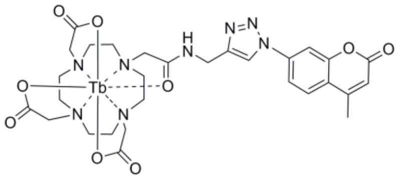<br><br>λ_{ex} = 345 nm	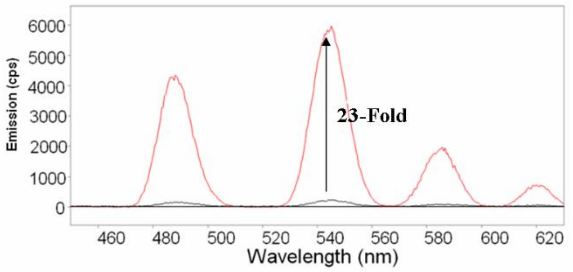
**19-Eu**<br>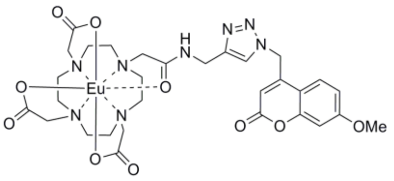<br><br>λ_{ex} = 325 nm	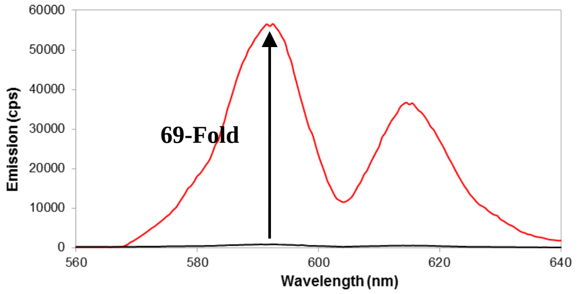
**19-Tb**<br>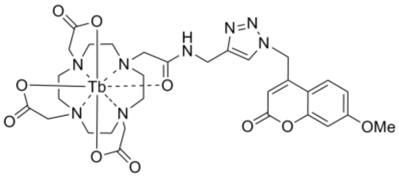<br><br>λ_{ex} = 325 nm	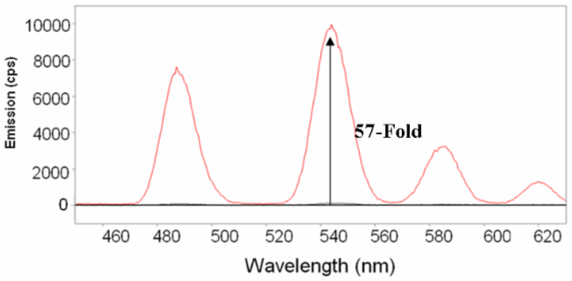

Table S3. Lanthanide luminescence picolinate complexes **18-Eu/Tb** and **19-Eu/Tb**; The black line is the control experiment of lanthanide-DOTA-alkyne and fluorophore azide (both 100 μM) in the absence of copper, the red line is 100 μM solution of crude lanthanide-triazole-fluorophore after CuAAC reaction. (time delay = 0.076 ms, slits = 10 nm, sample window = 5 ms, number of flashes = 20)

<table>
<tr><th>Triazole Complex</th><th>Luminescence response</th></tr>
<tr><td>

20-Eu

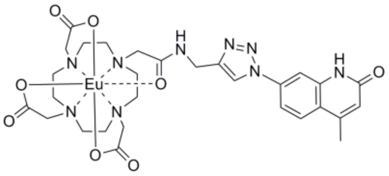

λex = 345 nm

</td><td>

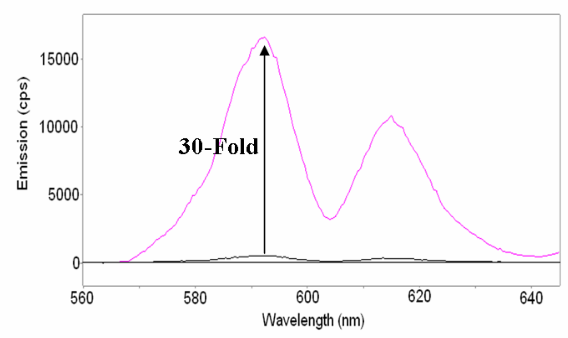

</td></tr>
<tr><td>

20-Tb

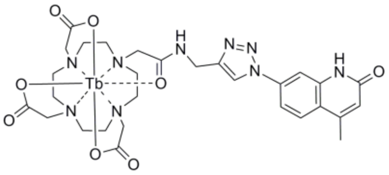

λex = 345 nm

</td><td>

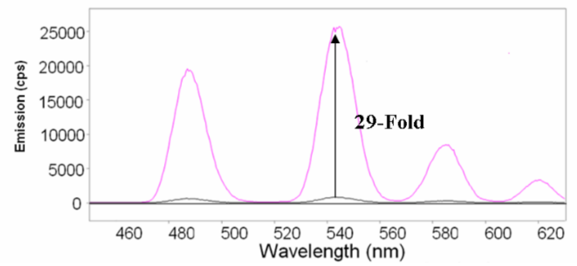

</td></tr>
<tr><td>

21-Tb

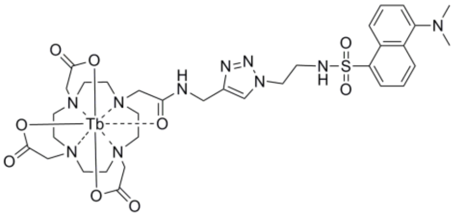

λex = 325 nm

</td><td>

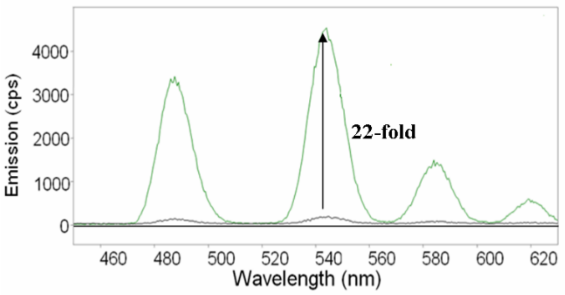

</td></tr>
</table>

Table S4. Lanthanide luminescence picolinate complexes **20-Eu/Tb** and **21-Tb**; The black line is the control experiment of lanthanide-DOTA-alkyne and fluorophore azide (both 100 μM) in the absence of copper, the pink/green line is 100 μM solution of crude lanthanide-triazole-fluorophore after CuAAC reaction. (time delay = 0.076 ms, slits = 10 nm, sample window = 5 ms, number of flashes = 20)

Figure S1: ESI mass spectrum for crude reaction product 20-Tb

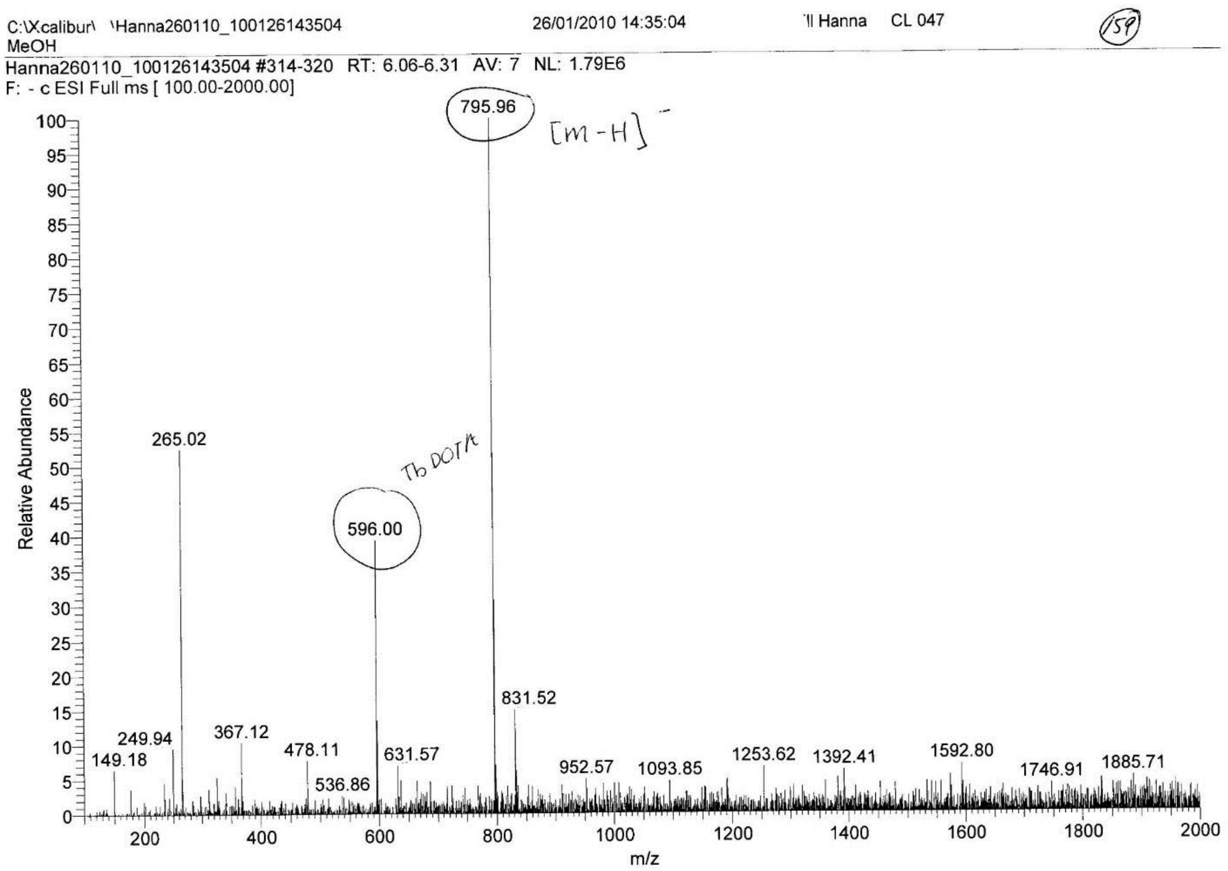

ESI-MS negative mode for the crude reaction product **20-Tb** (*m/z* $[M-H]^-$ 796) obtained after CuAAC reaction of a 1:1 mix of Tb-DOTA alkyne **1b** (*m/z* $[M-H]^-$ 596) and azide **12**.

Figure S2: HPLC chromatogram of crude reaction product 19-Tb

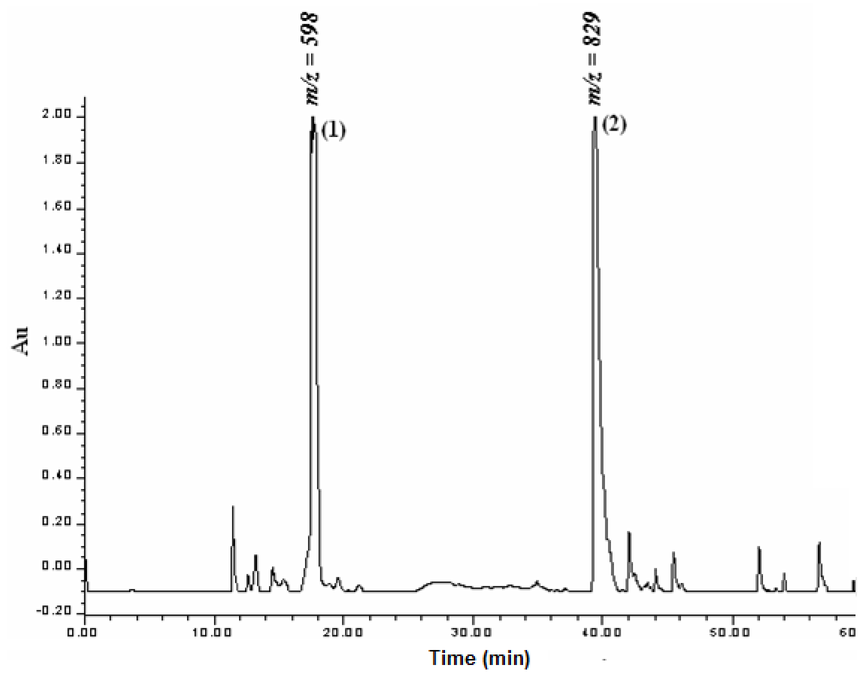

HPLC chromatogram of the crude reaction product **19-Tb** (*m/z* $[M+H]^+$ 829), obtained after CuAAC reaction of a 1:1 mix of alkyne **1b** (*m/z* $[M+H]^+$ 598) and azide **11**, annotated with the *m/z* values obtained for the major peaks post purification.

Normalised IR spectra for complexes 19 and 20:

Triazole Complex	Normalised IR
**19-Eu** 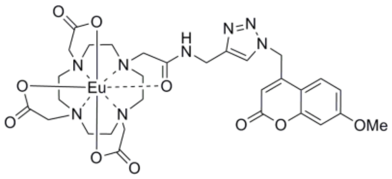 % Azide remaining at 2115/2115 cm^{-1} calculated as 16.1%	Normalised at 1612/1616 cm^{-1} 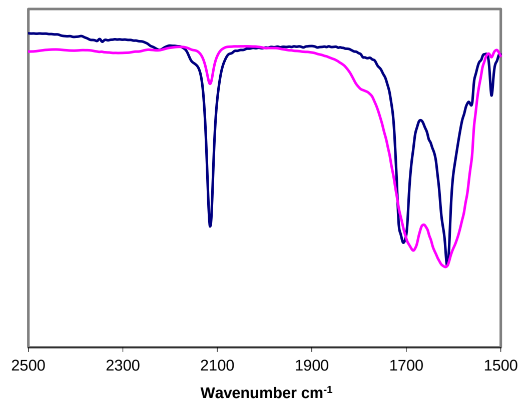
**19-Tb** 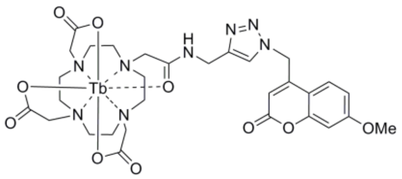 % Azide remaining at 2115/2114 cm^{-1} calculated as 15.3%	Normalised at 1614/1615 cm^{-1} 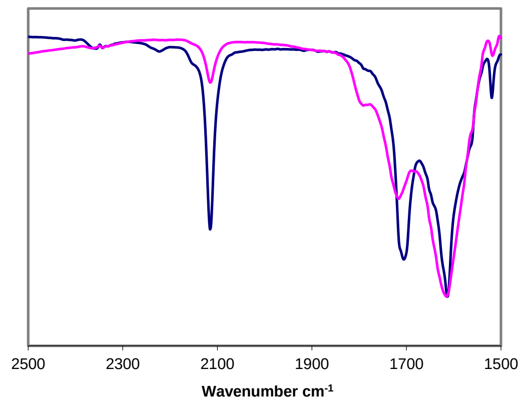

Table S5. FT-IR analysis of azide peak at 2100 cm^{-1} for crude sensors (pink) with normalisation of absorbance to carbonyl band in a 1:1 mixture of DOTA alkyne (**1a** or **1b**):fluorescent azide (**11**) (blue).

Triazole Complex	Normalised IR
**20-Eu**<br>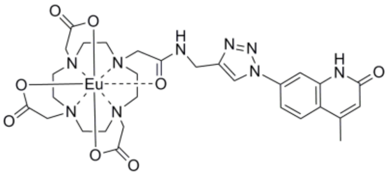<br>% Azide remaining at 2125/2121 cm^{-1} calculated as 17.0%	Normalised at 1628/1624 cm^{-1}<br>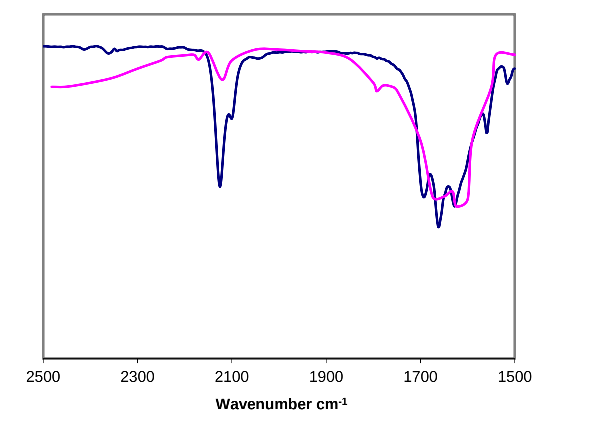
**20-Tb**<br>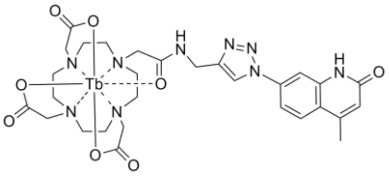<br>% Azide remaining at 2115/2120 cm^{-1} calculated as 21.6%	Normalised at 1614/1626 cm^{-1}<br>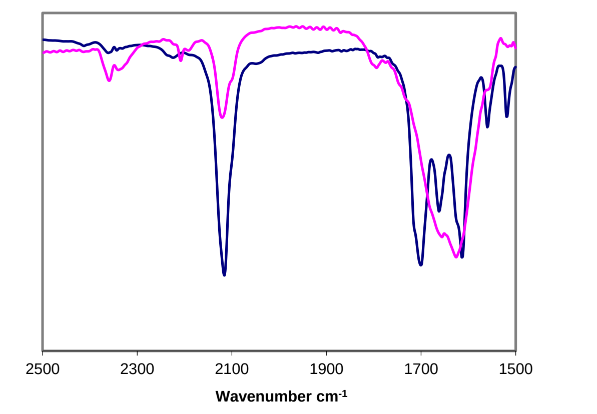

Table S6. FT-IR analysis of azide peak at 2100 cm^{-1} for crude sensors (pink) with normalisation of absorbance to carbonyl band in a 1:1 mixture of DOTA alkyne (**1a** or **1b**):fluorescent azide (**12**) (blue).

Compound 7 ^{1}H NMR (500 MHz, D_2O)

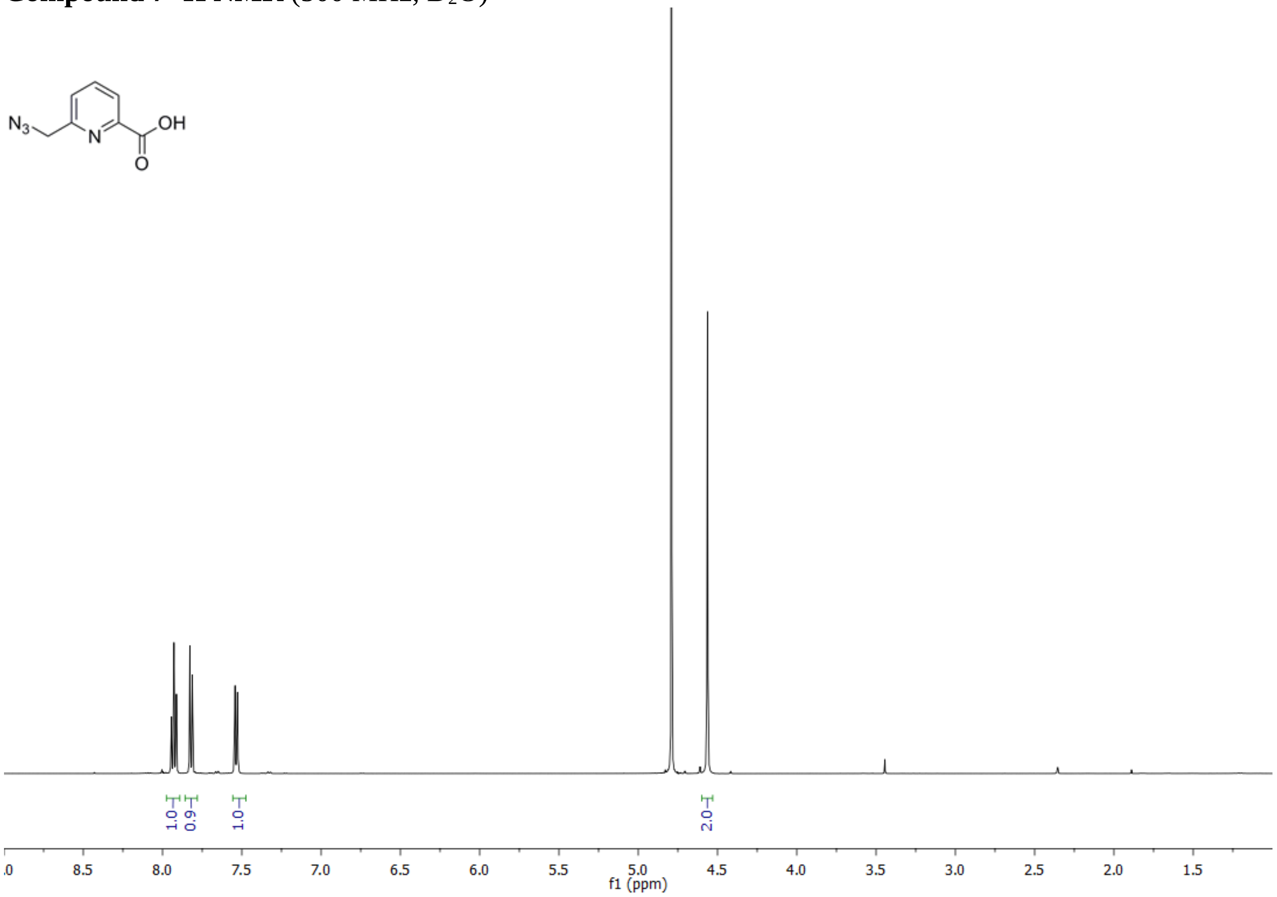

Compound 7 ^{13}C NMR (126 MHz, D_2O)

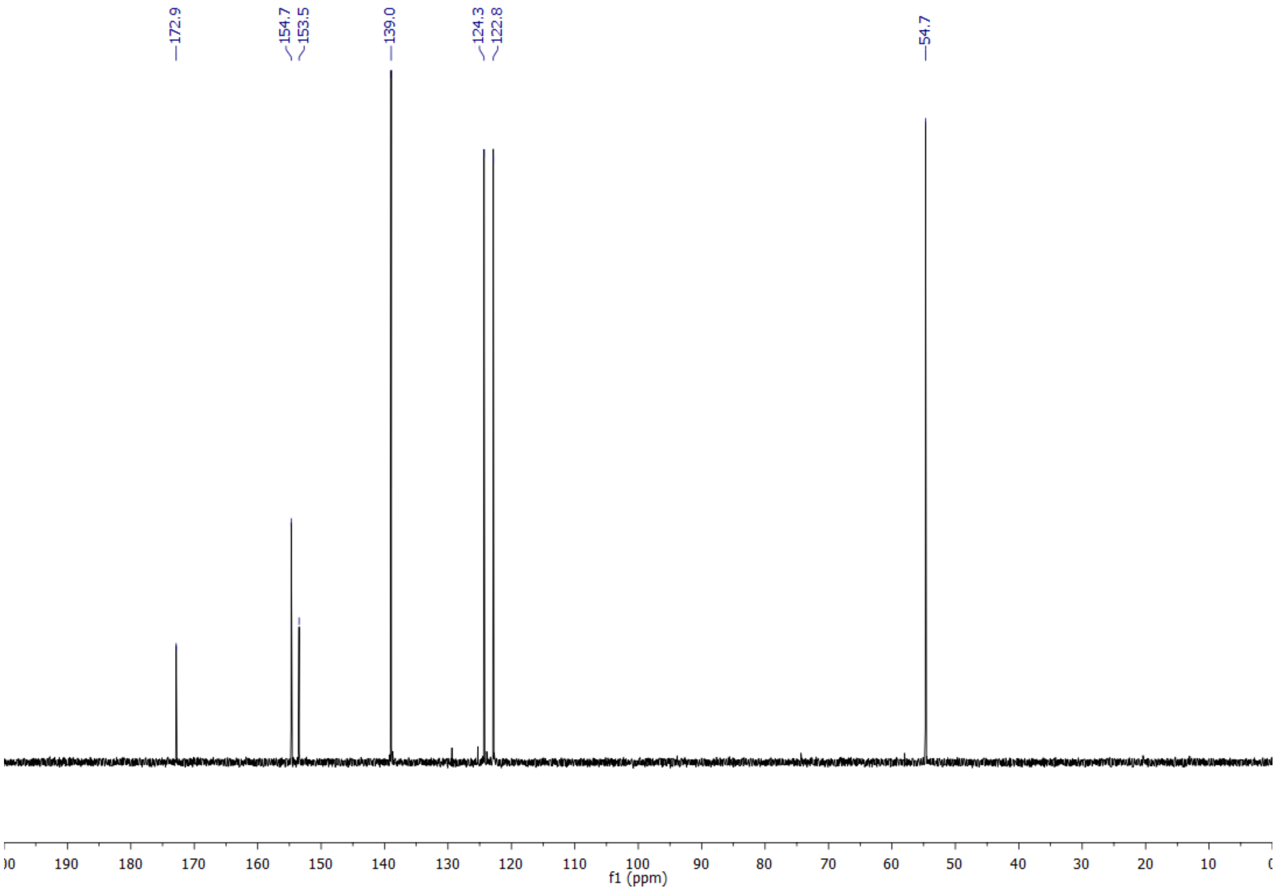

Compound 8 ^{1}**H NMR** (250 MHz, $CDCl_3$)

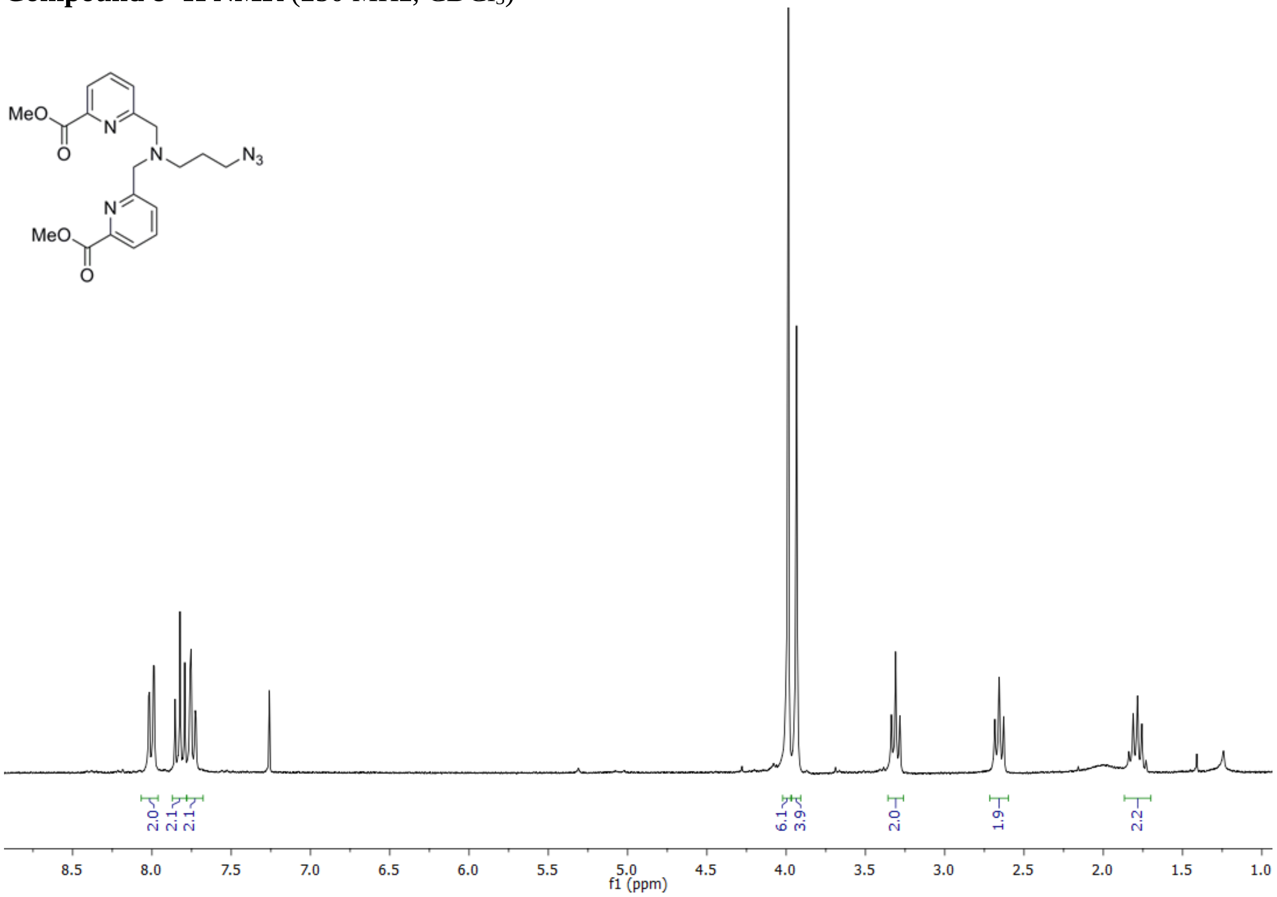

Compound 8 ^{13}**C NMR** (63 MHz, $CDCl_3$)

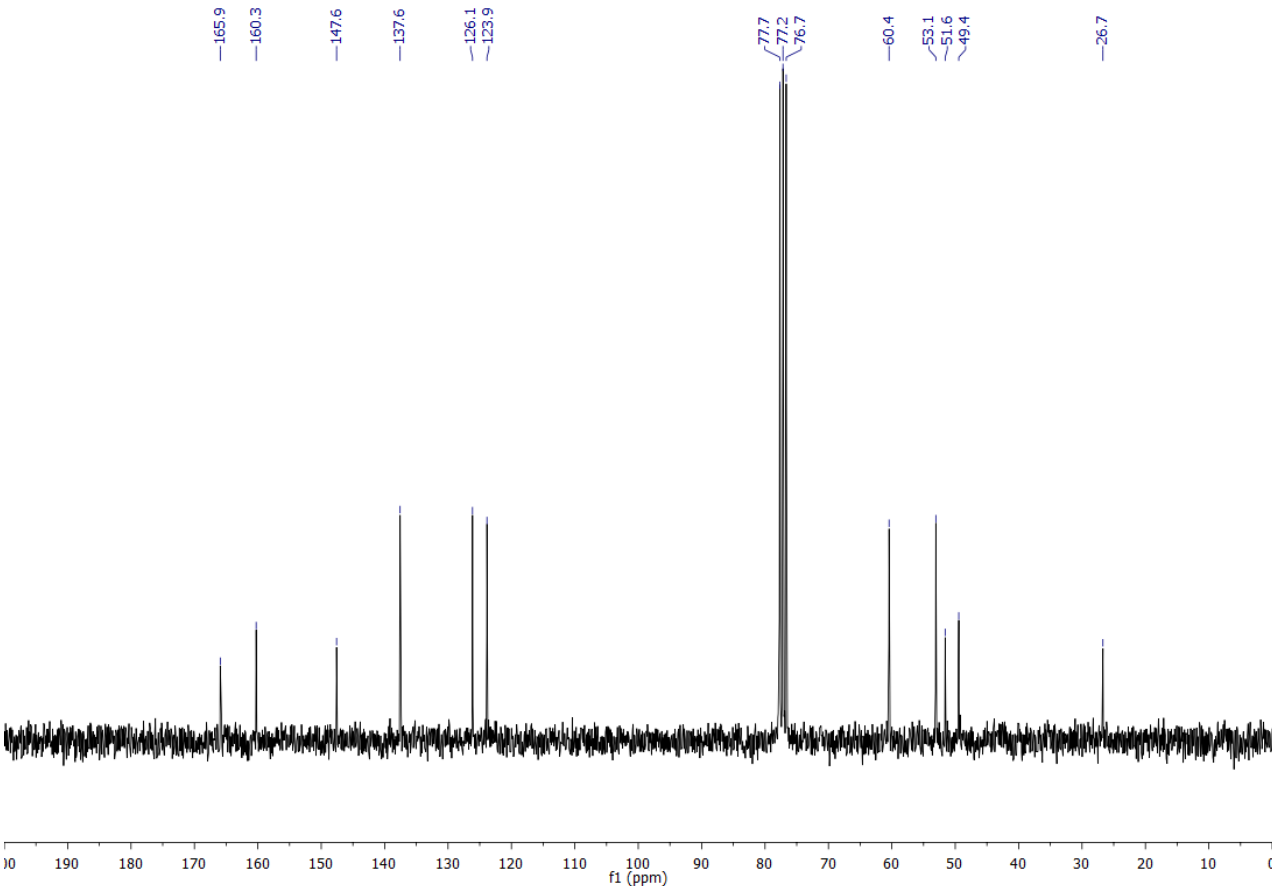

Compound 9 ^{1}H NMR (500 MHz, D_2O)

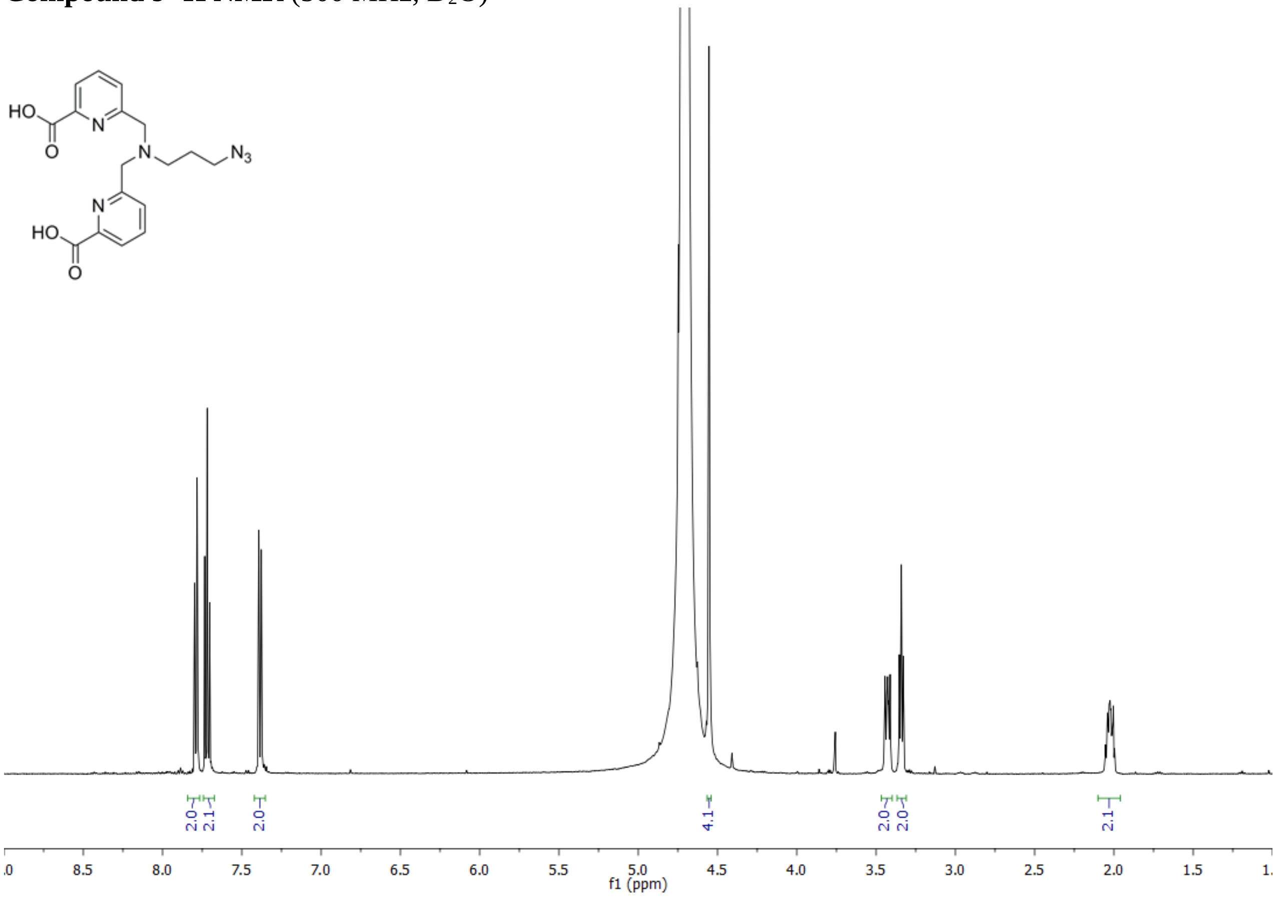

Compound 9 ^{13}C NMR (63 MHz, D_2O)

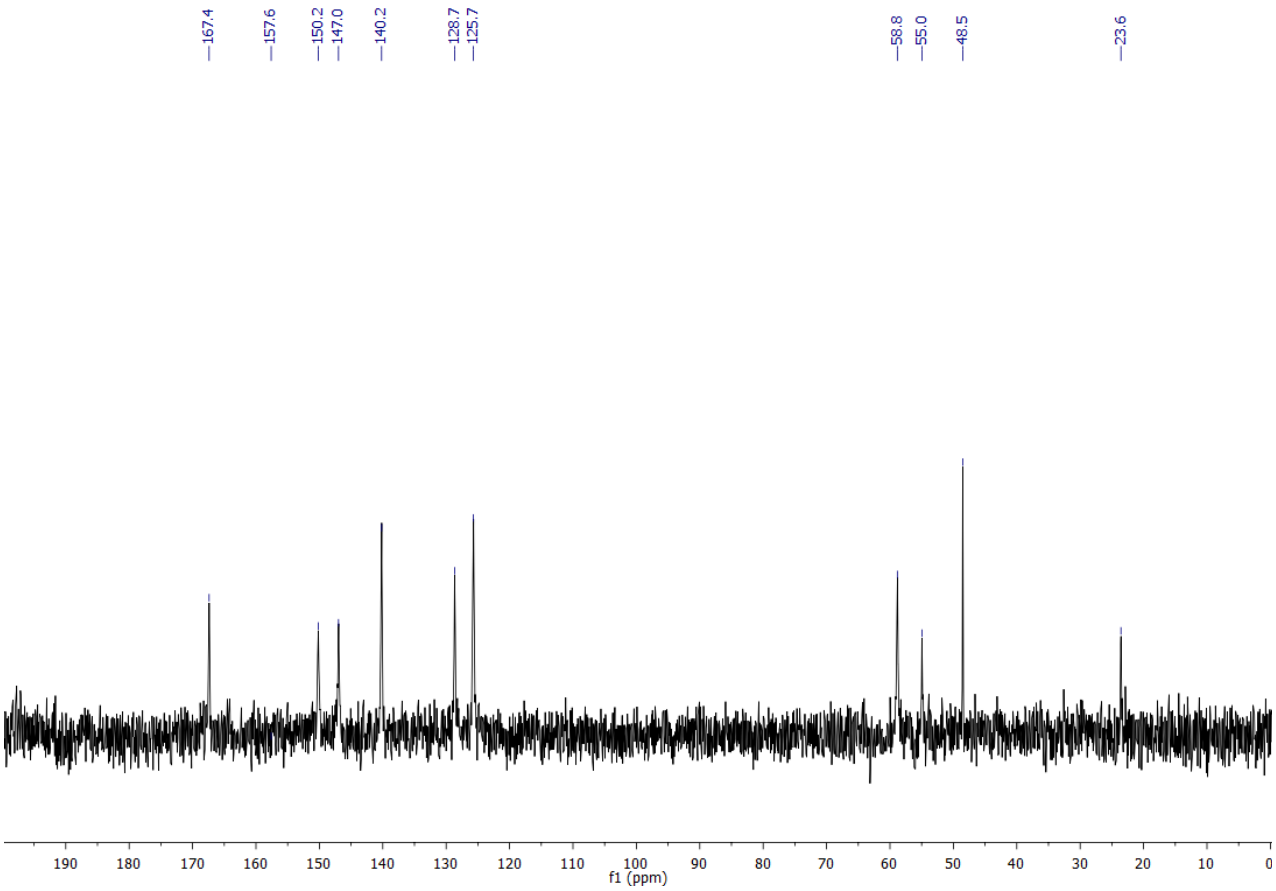

Figure S3: HPLC chromatogram of purified complex 19-Eu

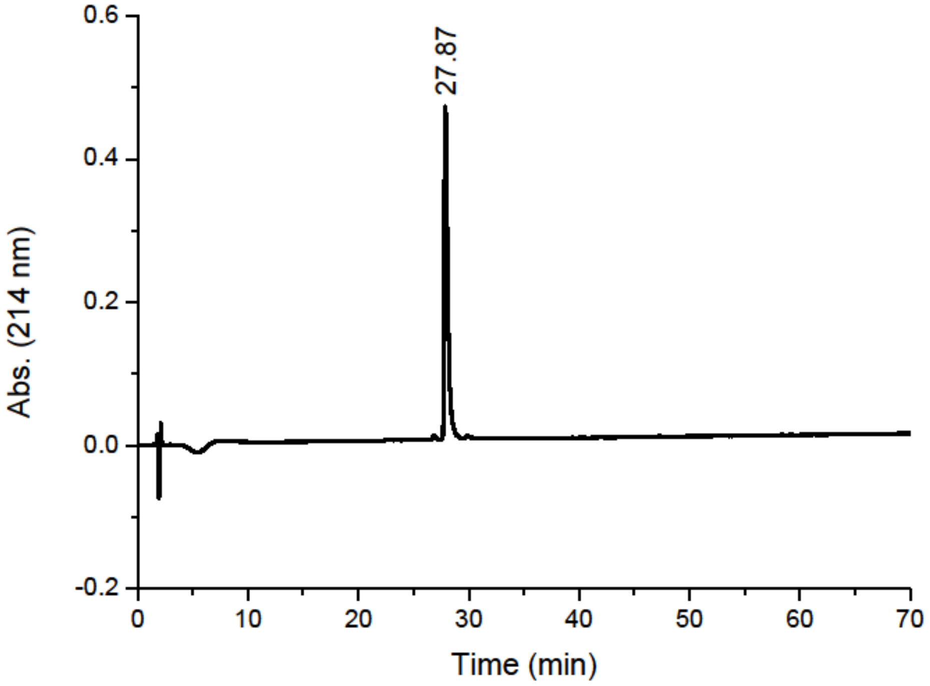

Analytical HPLC chromatogram of the purified complex **19-Eu** (*m/z* [M+H]$^+$ 823), obtained after CuAAC reaction of a 1:1 mix of alkyne **1a** and azide **11**.

Figure S4: Overlay of UV-vis spectra of purified complex 19-Eu and coumarin azide 11

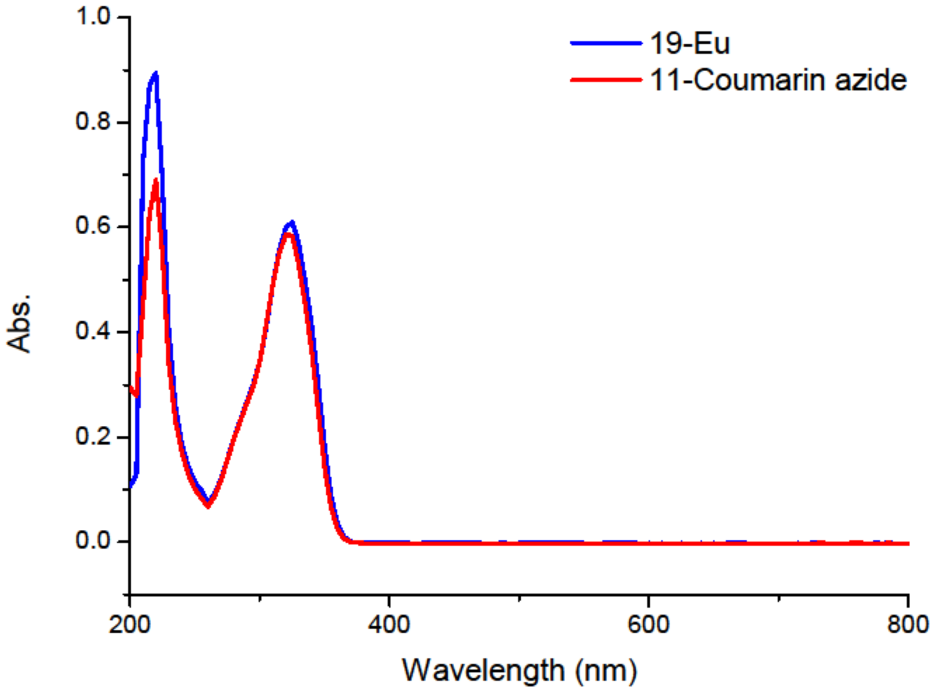

UV-vis data for the purified complex **19-Eu** and coumarin azide **11** were acquired on a Cary 50 spectrophotometer (Varian); scanned from 200-800 nm at standard concentrations of 50 µM in MeOH.

Figure S5: MS data for purified complex 19-Eu

(a) Full spectrum

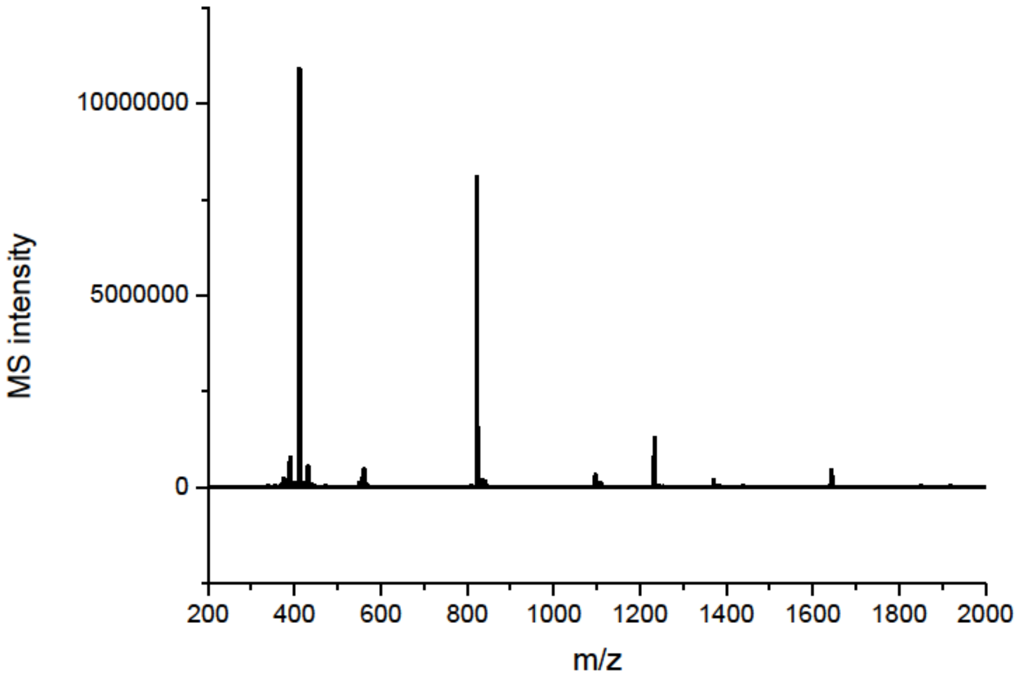

(b) [M+H]$^+$ peak expansion

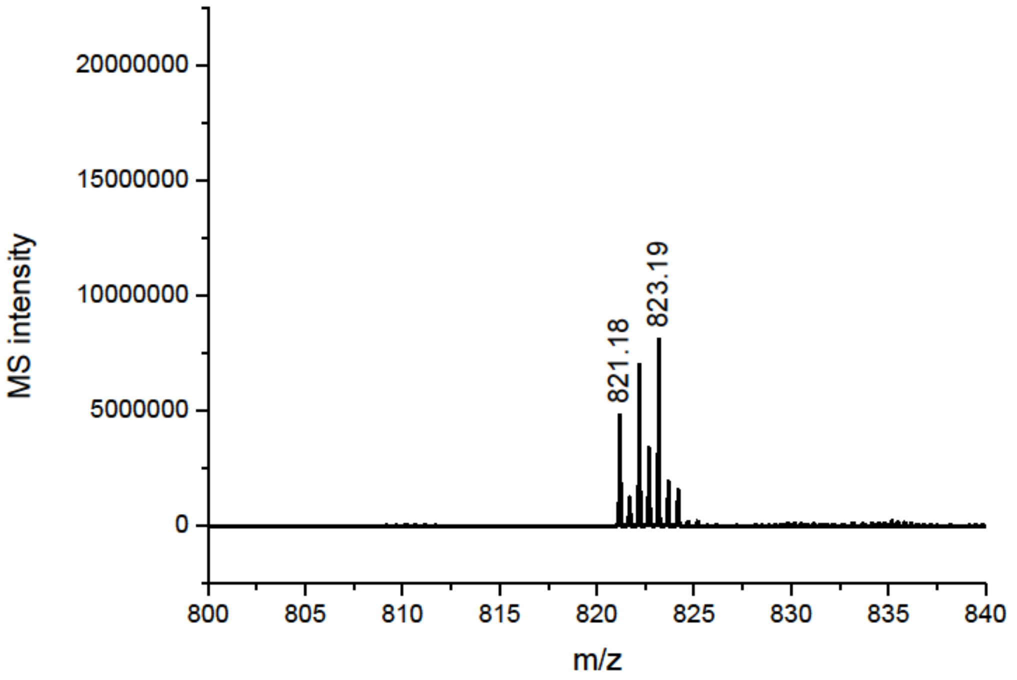

ESI-MS data was acquired using a Waters Aquity UPLC and SYNAPT G2 Q-TOF (A: H_2O + 0.1% formic acid, B: MeCN + 0.1 % formic acid; 5% → 95% (B) over 10 min; Flow 0.1 mL min^{-1}). Correction performed by injection of lockmass (Leucine-Enkephalin) at the start of each gradient.

Purified complex 19-Eu ^{1}H NMR (600 MHz, D_2O)

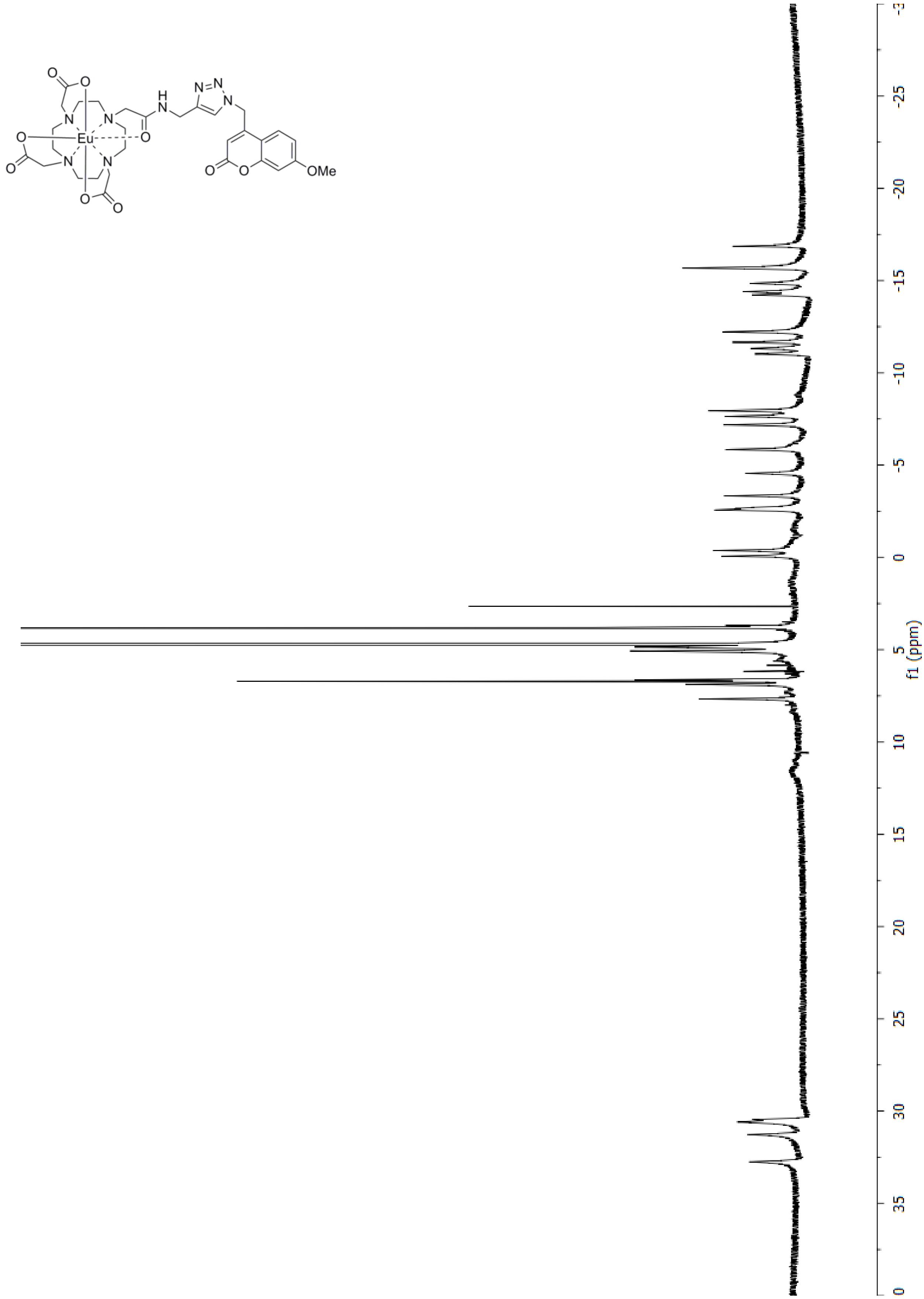

NMR data was acquired on a Bruker Avance 4-channel 600 MHz NMR Spectrometer, equipped with a TXI cryoprobe.

References:

[11] Viguier, R.F.H.; Hulme, A.N. A sensitized europium complex generated by micromolar concentrations of copper(I): Toward the detection of copper(I) in biology. *J. Am. Chem. Soc.*, **2006**, *128*, 11370-11371, DOI 10.1021/ja064232v.

[25] Axelsson, O.; Olsson, A. Synthesis of cyclen derivatives. *WO2006112723 A1* **2006**.

[26] Martinelli, J.; Balali-Mood, B.; Panizzo,R.; Lythgoe, M.F.; White, A.J.P.; Ferretti, P.; Steinke, J.H. G.; Vilar R. Coordination chemistry of amide-functionalised tetraazamacrocycles: structural, relaxometric and cytotoxicity studies. *Dalton Trans.* **2010**, *39*, 10056–10067, DOI 10.1039/C0DT00815J.

[36] Behera, H.; Ramkumar, V.; Madhavan, N. Cation-Transporting Peptides: Scaffolds for Functionalized Pores? *Chem. Eur. J.* **2015**, *21*, 10179-10184, DOI 10.1002/chem.201500881.

[37] Gracia, S.; Arrachart, G.; Marie, C.; Chapron, S.; Miguirditchian, M.; Pellet-Rostaing, S. Separation of Am (III) by solvent extraction using water-soluble H_4tpaen derivatives. *Tetrahedron*, **2015**, *71*, 5321-5336, DOI 10.1016/j.tet.2015.06.015.

[43] van Kalkeren, H.A.; Bruins, J.J.; Rutjes, F.P.J.T.; van Delft, F.L. Organophosphorus-catalysed Staudinger reduction, *Adv. Synth. Catal.* **2012**, *354*, 1417-1421, DOI 10.1002/adsc.201100967.

[44] Jawalekar, A.M.; Meeuwenoord, N.; Cremers, J.G.O.; Overkleeft, H.S.; van der Marel, G.A.; Rutjes, F.P.J.T.; van Delft, F.L. Conjugation of nucleosides and oligonucleotides by [3+2] cycloaddition. *J. Org. Chem.* **2008**, *73*, 287-290, DOI 10.1021/jo702023s.

[48] Kamaruddin, M.A.; Hossain, M.I.; Jarasrassamee, B.; Cheng, H.-C.; Ung, P.; O'Malley, W.; Thompson, P.; Graham, B.; Scanlon, D. A facile, click chemistry-based approach to assembling fluorescent chemosensors for protein tyrosine kinases. *Bioorg. Med. Chem. Lett.* **2011**, *21*, 329-331, DOI 10.1016/j.bmcl.2010.11.005.

[49] Maruani, A.; Alom, S.; Canavelli, P.; Lee, M.T.W.; Morgan, R.E.; Chudasama, V.; Caddick, S. A mild TCEP-based para-azidobenzyl cleavage strategy to transform reversible cysteine thiol labelling reagents into irreversible conjugates. *Chem. Commun.* **2015**, *51*, 5279-5282, DOI 10.1039/c4cc08515a.

[63] Zeng, X.; Coquiere, D.; Alenda, A.; Garrier, E.; Prange, T.; Li, Y.; Reinaud, O.; Jabin, I. Efficient synthesis of calix[6]tmpa: a new calix[6]azacryptand with unique conformational and host-guest properties. *Chem. Eur. J.* **2006**, *12*, 6393-6402, DOI 10.1002/chem.200600278.

[64] Mohamed, G. G.; El-Gamel, N. E. Structural, spectroscopic and thermal characterization of 2-*tert*-butylaminomethylpyridine-6-carboxylic acid methylester and its Fe(III), Co(II), Ni(II), Cu(II), Zn(II) and UO(2)(II) complexes. *Spectrochim. Acta A Mol. Biomol. Spectrosc.* **2005**, *61*, 1089-1096, DOI 10.1016/j.saa.2004.06.025.
